# Localization of the Carnation Italian ringspot virus replication protein p36 to the mitochondrial outer membrane is mediated by an internal targeting signal and the TOM complex

**DOI:** 10.1186/1471-2121-9-54

**Published:** 2008-09-23

**Authors:** Yeen Ting Hwang, Andrew W McCartney, Satinder K Gidda, Robert T Mullen

**Affiliations:** 1 Department of Molecular and Cellular Biology, University of Guelph, Guelph, Ontario, N1G 2W1, Canada; 2 JD Irving, Limited, Woodlands Division, 1350 Regent Street, Fredericton, New Brunswick, E3C 2G6, Canada

## Abstract

**Background:**

*Carnation Italian ringspot virus *(CIRV) is a positive-strand RNA virus that causes massive structural alterations of mitochondria in infected host cells, the most conspicuous being the formation of numerous internal vesicles/spherules that are derived from the mitochondrial outer membrane and serve as the sites for viral RNA replication. While the membrane-bound components of the CIRV replication complex, including a 36-kD RNA-binding protein (p36), are known to be essential for these changes in mitochondrial morphology and are relatively well characterized in terms of their roles in nascent viral RNA synthesis, how these proteins are specifically targeted and inserted into mitochondria is poorly defined.

**Results:**

Here we report on the molecular signal responsible for sorting p36 to the mitochondrial outer membrane. Using a combination of gain-of-function assays with portions of p36 fused to reporter proteins and domain-swapping assays with p36 and another closely-related viral RNA-binding protein, p33, that sorts specifically to the peroxisomal boundary membrane, we show that the mitochondrial targeting information in p36 resides within its two transmembrane domains (TMDs) and intervening hydrophilic loop sequence. Comprehensive mutational analysis of these regions in p36 revealed that the primary targeting determinants are the moderate hydrophobicity of both TMDs and the positively-charged face of an amphipathic helix within the intervening loop sequence. We show also using bimolecular fluorescence complementation (BiFC) that p36 interacts with certain components of the translocase complex in the mitochondrial outer membrane (TOM), but not with the sorting and assembly machinery (SAM).

**Conclusion:**

Our results provide insight to how viruses, such as CIRV, exploit specific host-cell protein sorting pathways to facilitate their replication. The characterization of the targeting and insertion of p36 into the mitochondrial outer membrane also sheds light on the mechanisms involved in sorting of host-cell membrane proteins to mitochondria, a process that has been largely unexplored in plants.

## Background

The hallmark of positive-strand RNA viruses is their ability to recruit distinct host-cell organelle membranes in order to create unique compartments at which viral RNA replication takes place [1-3]. This process is exemplified during tombusvirus infections of plant cells where, depending on the virus and host, peroxisomes or mitochondria undergo a series of remarkable structural rearrangements that ultimately results in their transformation into so-called multivesicular bodies (MVBs) [reviewed in [4,5]]. These novel structures form initially by the proliferation and progressive invagination of the organelle's boundary (outer) membrane, resulting in the matrix or intermembrane space containing hundreds of small (~80–150 nm diameter) vesicles and/or spherules which serve as the sites of viral RNA replication. MVBs often then form one or more large, vesicle/spherule-containing appendages that encircle portions of the neighbouring cytosol, yielding C-shaped or doughnut-shaped structures that frequently coalesce with other MVBs in the infected cell.

While the cytolopathological features of MVB biogenesis have been relatively well studied, many fundamental questions remain about the molecular mechanisms underlying the interaction of viral replication factors with host-cell membranes. For instance, the significance of the diversity of intracellular membranes used by different viruses is unknown. Likewise, the events involved in the specific intracellular targeting and membrane integration/assembly of the viral replication proteins, as well as the host-cell factors that facilitate these processes and/or mediate membrane remodelling are, in most cases, poorly studied and unclear.

CIRV is a member of the *Tombusviridae *family of positive-strand RNA plant viruses that include *Cymbidium ringspot virus *(CymRSV) and the *Tomato bushy stunt virus *(TBSV) [6,7]. Similar to other tombusviruses, the CIRV genome consists of a 4.8-kb linear, monopartite RNA molecule that contains five open reading frames (ORFs) [8], including ORF1 and ORF2, a 36-kD RNA-binding protein (p36) and its translation read-through product, a 95-kD RNA-dependent RNA polymerase (p95). Both p36 and p95 are the integral membrane-bound components of the virus' RNA replication complex and are located within the virus-induced vesicles/spherules of the MVB [9-12]. The remaining three ORFs in the CIRV genome encode a 41-kD coat protein, a 22-kD protein required for cell-to-cell movement of the virus, and a 19-kD protein that functions as a suppressor of virus-induced gene silencing [7].

In CIRV-infected cells, MVBs are derived from mitochondria [13] and the principal viral component involved in this process appears to be p36. For instance, analysis of full-length hybrid infectious clones of CIRV and CymRSV, which, unlike CIRV, recruits peroxisomes for its viral RNA replication, revealed that both of their ORF1s, namely p36 and the CymRSV 33-kD membrane-bound replication protein (p33), contain the determinants for the formation of MVBs derived from mitochondria or peroxisomes, respectively [14,15]. p36 expressed alone in either tobacco mesophyll or in yeast (*Saccharomyces cerevisiae*) cells is also sufficient to target the green fluorescent protein (GFP) to mitochondria and while these organelles are not entirely transformed into MVBs, they displayed dramatic alterations in their distribution and morphology, including proliferation of their outer membranes [16,17]. Interestingly, results from previous studies of p36 suggest also that its sorting to mitochondrial outer membranes is atypical since it does not appear to rely on mitochondrial-surface proteinaceous receptors nor a targeting/insertion signal similar to those typically found in most host-cell mitochondrial membrane- or matrix-localized proteins [18]. The nature of the sorting pathway for p36, instead, is considered to be complex, consisting of multiple, perhaps novel, targeting/insertion signals and a unique membrane insertion mechanism [18], however, this premise has not been experimentally tested.

Here, we describe the results of a comprehensive study of the molecular signals involved in the mitochondrial targeting of p36. We show using a combination of p36-reporter fusion proteins and p36-p33 hybrid proteins, wherein specific regions of p36 were replaced with those that constitute the peroxisomal targeting information in p33 (and vice versa), that the mitochondrial sorting of p36 is mediated by an internal targeting signal consisting of its two moderately hydrophobic TMDs and a positively-charged face of an amphipathic helix located within the intervening loop sequence. Notably, this targeting signal in p36 resembles the targeting determinants in several authentic mitochondrial outer membrane proteins from evolutionarily diverse organisms. We show also that p36 interacts with certain components of the translocase in the mitochondrial outer membrane (TOM), but not with the sorting and assembly machinery (SAM), in a manner consistent with the insertion of some host-cell mitochondrial outer membrane proteins. The implications of these findings in terms of CIRV's and other positive-strand RNA viruses' strategies to appropriate and subsequently modify mitochondrial sorting pathways to their own advantage are briefly discussed. Also discussed is how these results provide insight to the cellular processes underlying plant mitochondrial outer membrane protein sorting in general.

## Results and discussion

### p36 expressed transiently in tobacco BY-2 cells is localized to mitochondrial outer membranes

**Figure 1:**
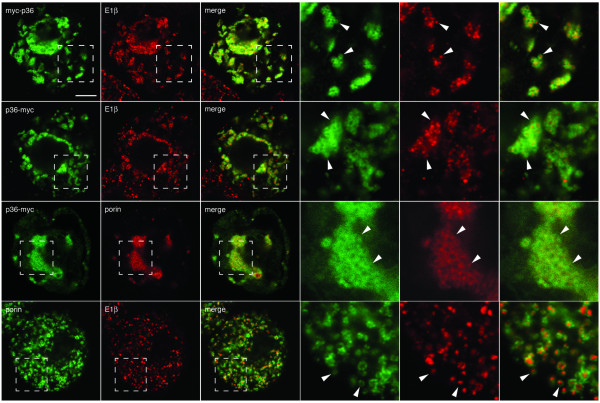
**Localization of p36 to the outer mitochondrial membrane in BY-2 cells**. Tobacco BY-2 cells were either non-transformed or transformed transiently (via biolistic bombardment) with either myc-p36 or p36-myc and then processed for immunofluorescence CLSM. Hatched boxes represent the portion of the cells shown at higher magnification in the panels to the right. Arrowheads indicate obvious examples of the torus fluorescent structures containing myc-p36 or p36-myc either delineating the spherical structures containing endogenous mitochondrial matrix-localized E1β or colocalizing with the torus structures containing the endogenous mitochondrial outer membrane protein porin. The bottom row of images show representative immunofluorescence staining patterns attributable to endogenous porin and E1β in non-transformed cells; arrowheads in the high magnified images indicate obvious examples of porin-containing torus structures (similar to those containing myc-p36 or p36-myc [compare with cells above]) enclosing the E1β-containing spheres. Bar = 10 μm.

To begin to decipher the intracellular targeting information within p36, we took advantage of tobacco (*Nicotiana tabacum*) Bright Yellow-2 (BY-2) suspension-cultured cells as a well-characterized *in vivo *import system [19-21]. Specifically, BY-2 cells were transiently transformed (via biolistic bombardment) with plasmid DNA encoding p36 fused at either its N or C terminus to the myc-epitope recognition motif (-EQKLISEEDL-; [22]) and, then, following a 4 h incubation period to allow for gene expression and protein sorting, cells were processed for indirect immunofluorescence confocal-laser scanning microscopy (CLSM). As shown in Figure 1, both transiently-expressed myc-p36 and p36-myc localized exclusively to globular structures that were found mostly within the perinuclear region of the cell and contained endogenous E1β, a protein subunit of the pyruvate dehydrogenase complex located in the mitochondrial matrix [23]. Interestingly, close inspection of these p36-containing globular structures at higher magnification revealed that they actually consisted of numerous torus or donut-shaped structures that contained myc-p36 or p36-myc and that enclosed spherical fluorescent structures containing matrix-localized E1β (refer to arrowheads in Figure 1). These toroidal structures also contained the endogenous mitochondrial outer membrane protein porin [24], as evidenced by the colocalization of p36-myc and porin (Figure 1). Taken together, these results indicate that p36 sorts to mitochondrial outer membranes in BY-2 cells and that the expression of this viral protein causes mitochondria to coalesce in perinuclear regions, in contrast to mitochondria in non-transformed cells that are distributed throughout the cytosol; refer to images of the immunostaining of endogenous mitochondrial porin and E1β in the non-transformed cells shown in the bottom row of Figure 1.

While it remains to be determined whether the aggregated mitochondria in p36-transformed cells were modified also in terms of their ultrastructure, this possibility is likely since expression of p36 (e.g., p36-GFP) in various other cell types (e.g., leaf mesophyll protoplasts and yeast cells) led to similar aggregations of mitochondria, as well as a proliferation of their outer membranes [16-18]. Indeed, high-magnified views of myc-p36- or p36-myc-transformed BY-2 cells in this study revealed that the immunostaining pattern attributable to endogenous porin in these cells was more diffuse than that observed in non-transformed cells (Figure 1), suggesting that the outer mitochondrial membranes in the former cells were altered in terms of their morphology. The nature of these changes or the mechanism(s) by which p36 participates in this process were not, however, examined further.

The localization of myc-p36 and p36-myc to mitochondria indicates also that appending the myc epitope to the N or C terminus of p36 did not disrupt the protein's normal targeting behaviour, since non-tagged (wild-type) p36 colocalized also with endogenous porin in the outer membranes of aggregated mitochondria (refer to Additional file 1A, top row). p36 was immunodetected in these cells using polyclonal antibodies raised against a synthetic peptide corresponding to an amino acid sequence in the C-terminal half of TBSV p33 and p92 replicase proteins (residues 184–203; [25]) and conserved in CIRV p36 and p95 (residues 218–237). However, due to limited availability of this antibody reagent, only myc-tagged versions of p36, primarily p36-myc, were employed in the remainder of the experiments described in this manuscript.

The localization of epitope-tagged and wild-type p36 to mitochondrial outer membranes, and their subsequent effects on mitochondrial morphology and distribution, were similar also to when p36 was co-expressed with its allied replication protein, p95, either together alone (Rep) or together in the context of full-length CIRV, i.e., a CIRV cDNA positioned within a plant expression plasmid (Additional file 1A). Confirmation of the infectivity of this CIRV cDNA was that *Chenopodium quinoa *leaves 7 to 10 days after rub-inoculation displayed local lesions (Additional file 1B) that resembled the disease symptoms reported for leaves infected with native CIRV RNA [4]. Moreover, electron microscopic analyses of these CIRV cDNA-inoculated leaf samples revealed the presence of mitochondrial-derived MVBs that were not observed in cells from mock-transformed leaves (Additional file 1C). The results for the mitochondrial localization of p36 expressed in the context of full-length CIRV (or Rep) are important because they indicate that, compared to the expression of p36 alone (either wild-type or epitope tagged), the viral protein behaves in a similar manner in terms of its intracellular localization. Thus, we deemed it appropriate to study the mitochondrial targeting information of p36 when expressed on its own.

### p36 is orientated in mitochondrial outer membranes in an N_out_-C_out _topology

The topological orientation of p36 in mitochondrial outer membranes was assessed using a differential detergent permeabilization assay [26]. Specifically, BY-2 cells were transformed transiently with N- or C-terminal myc-tagged versions of p36 and then fixed and incubated either with triton X-100, which perforates all cellular membranes, or with digitonin, which perforates only the plasma membrane.

**Figure 2:**
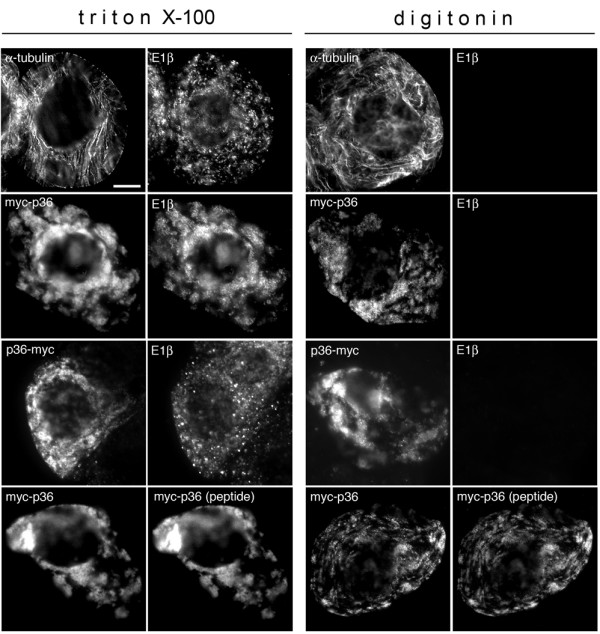
**Topological orientation of p36 in differentially permeabilized BY-2 cells**. BY-2 cells were either non-transformed or transformed transiently (via biolistic bombardment) with myc-p36 or p36-myc, fixed, and then permeabilized with either triton X-100 (which permeabilizes both the plasma membrane and organellar membranes) or digitonin (which permeabilizes only the plasma membrane). Permeabilized cells were then processed for (immuno)epifluorescence microscopy using antibodies raised against (as indicated by the labelling at the top left of each micrograph) either cytosolic α-tubulin, mitochondrial matrix-localized E1β, the myc epitope and/or the p36 C-terminal peptide sequence (amino acids 218–237) located downstream of the protein's second (of two) predicted TMDs. Bar = 10 μm.

As shown in Figure 2 (top row), control experiments with myc-p36-transformed cells verified that permeabilization with triton X-100 allowed for the immunodetection (via epifluorescence microscopy) of endogenous proteins within the cytosol (i.e., α-tubulin) and within subcellular compartments (i.e., E1β within the mitochondrial matrix), whereas permeabilization with digitonin allowed for the immunodetection of only cytosolic proteins (i.e., α-tubulin, but not E1β). Figure 2 shows also that when myc-p36- or p36-myc-transformed cells were permeabilized with either triton X-100 or digitonin both expressed proteins were immunodetected, whereas endogenous E1β was only immunodetected in the corresponding triton-X-100-permeabilized cells. Likewise, myc-p36 was immunodetected in both triton X-100- and digitonin-permeabilized cells that were incubated with antibodies specific for myc or the p36 C-terminal peptide mentioned above, i.e., amino acid residues 218–237 located downstream of the second (of two) predicted TMDs in p36.

Overall, these data are in agreement with the previously proposed model for the membrane topology of p36 based on the hydropathy profile of the protein's primary amino acid sequence [15] and protease resistance analyses of p36 in either CIRV-infected plants or isolated mitochondria [18]. In this model, p36 is predicted to be an integral membrane protein that contains two TMDs (residues 102–119 and 166–188), an intervening loop region (residues 120–165) orientated towards the mitochondrial intermembrane space, and N- and C terminal portions of the protein (residues 1–101 and 189–296) that face the cytosol. Unfortunately, our efforts to confirm the inward orientation of the intervening loop sequence in p36 were unsuccessful, because addition of the myc-epitope sequence to this portion of the protein caused the resulting mutant to be mislocalized from mitochondria to the cytosol in transformed cells (data not shown), a result that is likely due to the fact that, as discussed below, the loop sequence of p36 contains essential mitochondrial targeting information that was disrupted by the addition of the myc-epitope motif.

### An internal domain of p36 composed of two TMDs and an intervening loop sequence functions as a mitochondrial targeting signal

**Figure 3:**
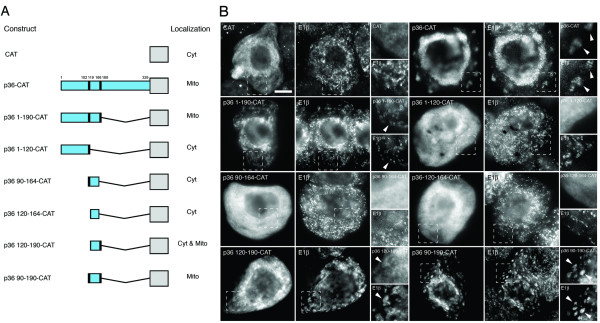
**Localization of p36-CAT fusion proteins in BY-2 cells**. **(A) **Schematic illustrations of CAT and various p36-CAT fusion proteins and their corresponding subcellular localizations in transformed (via biolistic bombardment) BY-2 cells. The numbers in the name of each fusion construct denote the specific amino acid residues from p36 that were fused to the N terminus of CAT. Portions of the p36 ORF are colored blue or black, the latter denoting the two putative TMDs in p36 and the numbers shown above p36-CAT include the position of each TMD's left and right amino acid border. Grey boxes denote CAT. Mito, mitochondria; Cyt, cytosol. **(B) **Representative (immuno)epifluorescence micrographs illustrating the localizations of the various constructs shown in (A). Each micrograph is labelled at the top left with the name of the transiently-expressed fusion protein or the endogenous mitochondrial marker protein, E1β. Hatched boxes represent the portion of the cells shown at higher magnification in the panels to the right. Arrowheads indicate obvious colocalizations. Bar = 10 μm.

**Figure 4:**
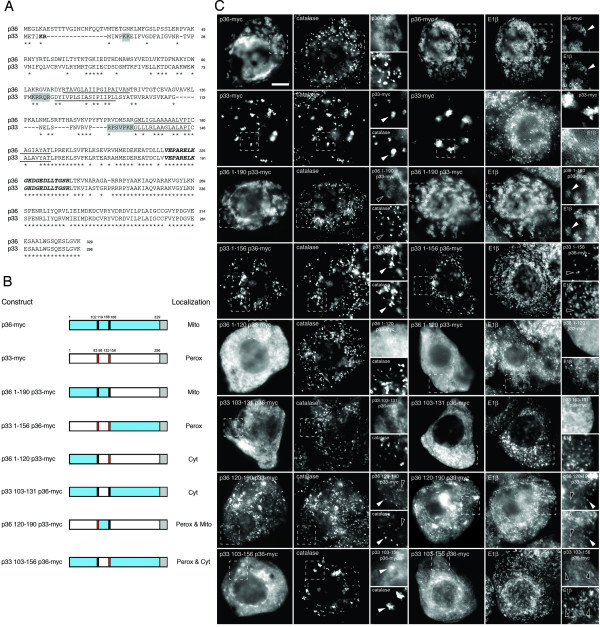
**Localization of p36-p33 hybrid proteins in BY-2 cells**. **(A) **Alignment of the deduced amino acid sequences of CIRV p36 and TBSV p33. Sequences were obtained from GenBank and aligned using the ClustalW . Identical amino acids in each protein are indicated with asterisks and putative TMDs (underlined) were determined using TMHMM (version 2.0) and visual inspection. Clusters of amino acids in TBSV p33 that were reported previously [25] to constitute the peroxisomal (i.e., -K_11_K_12_-, K_76_RRQR_80_- and -R_124_PSVPKK_130_-) or pER (i.e., -K_5_R_6_-) targeting signals are shaded grey or bolded, respectively. Conserved residues in p33 (residues 184–203) and p36 (residues 218–237) that are immunorecognized by a polyclonal antibody raised against this (synthetic) peptide sequence [25] are italized and bolded. **(B) **Schematic illustrations of C-terminal myc-tagged p36 (p36-myc), p33 (p33-myc) and various p36-p33 hybrid proteins and their corresponding subcellular localizations in transformed (via biolistic bombardment) tobacco BY-2 cells. The numbers in the name of each hybrid construct denote the specific amino acid residues from p36 or p33 that were fused to (trunctated) p33-myc or p36-myc, respectively. Portions of the p36 ORF are colored blue or black, the latter denoting the two putative TMDs in p36 and the numbers shown above p36-myc indicating their relative amino acid positions. Similarly, portions of the p33 ORF are colored white and red, the latter denoting the two putative TMDs in p33 and the numbers shown above p33-myc indicating their relative amino acid positions. The grey colored box for each construct denotes the C-terminal appended myc epitope. Cyt, cytosol; Mito, mitochondria; Perox, peroxisome. **(C) **Representative (immuno)epifluorescence micrographs illustrating the localizations of the various constructs shown in (B). Each micrograph is labelled at the top left with the name of either the transiently-expressed protein, the endogenous mitochondrial matrix marker protein E1β, or the endogenous peroxisomal matrix marker protein catalase. Hatched boxes represent the portion of the cells shown at higher magnification in the panels to the right. Solid arrowheads indicate obvious colocalizations; open arrowheads indicate obvious non-colocalizations. Bar = 10 μm.

While previous studies using chimeras of CIRV p36 and CymRSV p33, expressed either in the context of the full-length virus or alone, revealed that the N terminal half of each protein is responsible for its sorting to mitochondria and peroxisomes, respectively [14,15], results from subsequent mutational analyses of p36 expressed alone provided only limited insight to the precise nature the protein's mitochondrial targeting information [18]. For instance, based on the targeting results of various p36 deletion mutants, Weber-Lotfi et al. [18] concluded that the mitochondrial sorting of p36 is mediated by multiple determinants located within a relatively large portion of the protein's N-terminal half, i.e., residues 84–196 which includes both of the protein's TMDs and their flanking regions, and that together these determinants might function cooperatively as a so-called signal loop-anchor type mitochondrial targeting sequence. These authors also speculated that this putative signal loop-anchor targeting sequence for p36 was unique because, rather than being comprised of non-continuous structural elements and/or three-dimensional folding features, such as those that typically constitute the targeting determinants in host-cell multi-spanning mitochondrial outer membrane proteins with β-barrel structures, the mitochondrial targeting determinants in p36 (a non-β-barrel protein) were part of a linear stretch of the protein. The physicochemical properties of these potentially novel targeting determinants in p36 were not, however, defined. Thus, to better understand the mitochondrial targeting information in p36 we carried out a comprehensive mutational analysis of the protein, employing initially both gain-of-function targeting assays with different portions of p36 fused to a reporter protein (Figure 3) and domain-swapping assays with p36 and p33 (Figure 4).

As shown in Figure 3A, gain-of-function targeting experiments were carried out using a series of fusion proteins that consisted of p36, or portions thereof, appended to the bacterial passenger protein chloramphenicol acetyltransferase (CAT) and that were expressed transiently in BY-2 cells. The representative micrographs presented in Figure 3B show that while CAT expressed alone accumulated throughout the cytosol, p36-CAT, consisting of full-length p36 fused to the N terminus of CAT, localized exclusively to aggregated mitochondria. These latter data are consistent with the localization of myc-tagged and wild-type p36 to (aggregated) mitochondria in BY-2 cells (refer to Figure 1 and Additional file 1), as well as p36 fused to GFP and expressed transiently (via agrobacterium infiltration) in tobacco leaf mesophyll cells [17,18], and indicate that the CAT moiety used here as a reporter passenger protein does not alter the sorting behaviour of p36.

Similar to p36-CAT, p36 1-190-CAT, containing residues 1–190 of p36 including the protein's N-terminal hydrophilic domain, TMDs 1 and 2, and the intervening loop sequence appended to CAT, localized to (aggregated) mitochondria (Figure 3B), confirming also the results presented previously by Weber-Lotfi et al. [18] that the N-terminal half of the protein contains its mitochondrial targeting information. By contrast, p36 1-120-CAT, which includes only the N-terminal hydrophilic domain and TMD1 of p36, accumulated in the cytosol. Notably, the mitochondria in these p36 1-120-CAT-transformed cells, similar to mitochondria in cells transformed with CAT alone, were not altered in terms of their morphology and/or distribution (compare endogenous E1β immunostaining in p36 1-120-CAT-and CAT-transformed cells; Figure 3B), and reinforcing the notion that this fusion protein was not targeted to mitochondria.

Other p36-CAT fusion proteins comprised of smaller portions of the p36 N-terminal half also did not target to mitochondria. That is, both p36 90-164-CAT and p36 120-164-CAT, consisting of TMD1 and the intervening loop sequence and the intervening loop sequence alone, respectively, localized to the cytosol (Figure 3B). On the other hand, both p36 120-190-CAT and p36 90-190-CAT localized to (aggregated) mitochondria, albeit the former fusion protein only did so in a relatively inefficient manner since it accumulated also in the cytosol (Figure 3B). Based on the results presented here (Figure 3), p36 90-190-CAT contains the minimally sufficient portion of the protein capable of efficiently targeting CAT to mitochondria and, similar to wild-type p36, is localized to the outer membranes of mitochondria in an N_out_-C_out _topology (refer to Additional files 2A and 2B for high-magnification CLSM and differential permabilization assays with p36 90-190-CAT).

Characterization of the mitochondrial targeting information in p36 was also carried out using domain-swapping assays with TBSV p33, an ortholog of CymRSV that has been recently well characterized in terms of its peroxisomal membrane targeting signal [25]. Alignment of the deduced amino acid sequences for p36 and p33 revealed that while these proteins are nearly identical in their C-terminal halves, they are significantly divergent with respect to their N termini (Figure 4A). For instance, although both proteins are predicted to contain two TMDs located at relatively similar positions within their N-terminal halves (underlined, Figure 4A), p36 also possesses a number of amino acid residues in this region of the protein that are not found in p33. The most conspicuous of these being stretches of unique residues located near the extreme N terminus and within the intervening loop sequence of p36 (Figure 4A). Alignment of the p36 and p33 sequences revealed also that the multiple targeting signal motifs responsible for sorting nascent p33 initially to peroxisomes (i.e., -K_11_K_12_-, K_76_RRQR_80_- and -R_124_PSVPKK_130_-) (shaded in grey in Figure 4A) and then from peroxisomes to a subdomain of the endoplasmic reticulum (ER) referred to as peroxisomal ER (pER) (i.e., -K_5_R_6_-) (bolded in Figure 4A) [25] are not conserved in p36, consistent with the apparently distinct intracellular sorting pathways employed by these two proteins.

As shown in Figures 4B and 4C, p36-myc and p33-myc (TBSV p33 appended to a C-terminal myc-epitope tag) sorted exclusively to aggregated mitochondria and peroxisomes, respectively, in transiently-transformed BY-2 cells. The aggregation of peroxisomes in p33-myc-transformed cells is apparent by comparing the immunostaining pattern attributable to the endogenous peroxisomal matrix enzyme catalase in these cells with that of p36-myc-transformed cells, wherein catalase-containing peroxisomes display a normal (punctate) morphology and distribution (Figure 4C). These data for the localization of p33-myc to altered peroxisomes was expected, since previously published results with wild-type (non-tagged) p33 expressed for the same length of time (i.e., 4 h following biolistic bombardment) revealed that it was localized exclusively to peroxisomes and caused the organelles to coalesce [25]. Also similar to wild-type p33 [25], p33-myc sorted from peroxisomes to pER at later time points following bombardment (i.e., 24 h) (data not shown), indicating that the myc epitope also did not disrupt the normal peroxisome-to-pER sorting of the protein. Nevertheless, in order to minimize any potential sorting complexities associated with the targeting of p33 from peroxisome to pER at later time points, all of p33/p36-myc hybrids employed in domain-swapping assays were assessed only at 4 h post-bombardment for peroxisomal versus mitochondrial targeting.

Replacement of the N-terminal half of p33 (residues 1–156) with the corresponding sequences from p36 (residues 1–190), including the N-terminal hydrophilic domain and both TMDs plus the intervening loop sequence, resulted in the hybrid protein (p36 1–190 p33-myc) being sorted to aggregated mitochondria, but not to peroxisomes (Figure 4C). Conversely, p33 1–156 p36-myc, consisting of the N-terminal half of p33, including all three of the protein's peroxisomal targeting signals (i.e., -K_11_K_12_-, K_76_RRQR_80_- and -R_124_PSVPKK_130_-; refer to Figure 4A) [25], fused to the C-terminal half of p36, sorted exclusively to peroxisomes in a manner similar to full-length p33-myc (Figure 4C).

Figures 4B and 4C show also that fusion of the N-terminal 120 amino acids of p36 (including the protein's N-terminal hydrophilic domain and first TMD) to the remaining C-terminal portion of p33-myc resulted in the hybrid protein (p36 1–120 p33-myc) being localized to the cytosol, a result similar to when this N-terminal region of p36 was fused to CAT (p36 1-120-CAT) (Figure 3). Localization to the cytosol was observed also when the intervening loop sequence from p36 was replaced with the loop sequence from p33 (p33 103–131 p36-myc), indicating that this region of p36 contains essential mitochondrial targeting information. In accordance with this premise, the p36 intervening loop sequence together with its second TMD was capable of redirecting, albeit inefficiently, p33-myc to mitochondria, i.e., p36 120–190 p33-myc was sorted to both mitochondria and peroxisomes (Figure 4C), and further showing that this hybrid protein contains targeting information for both organelles. By contrast, mitochondrial targeting of p36 was completely abolished when its intervening loop sequence and TMD2 was replaced with the corresponding sequences from p33 (residues 103–156), i.e., p33 103–156 p36-myc was not localized to mitochondria, but instead localized to both peroxisomes and the cytosol (Figure 4C). The apparent inefficient sorting of this hybrid protein to peroxisomes is likely due to the fact that it possessed only one of the three peroxisomal targeting signals within p33, i.e., -R_124_PSVPKK_130_- (refer to p33 sequence shown in Figure 4A).

Overall, the data presented in Figures 3 and 4 confirm and extend the observations of Weber-Lotfi et al. [18] that the minimally sufficient mitochondrial targeting information in p36 is located within protein's N-terminal half, including both TMDs and the intervening loop sequence. Additional flanking sequences immediately upstream and downstream of TMD1 and TMD2, respectively, are not essential, however, for targeting p36 to mitochondria. For instance, the N-terminal hydrophilic portion of p36 was insufficient in sorting CAT (Figure 3) or a p36/p33 hybrid protein containing the remaining C terminal portion of p33 (Figure 4) to mitochondria. Thus, our data contradict the previously proposed notion that the N-terminal hydrophilic domain of p36, specifically, the residues immediately upstream of TMD1, contain a distinct mitochondrial targeting determinant [18]. It appears instead that p36 contains only one mitochondrial targeting signal: a signal that consists of a relatively large portion of the protein (residues 90–190) and includes several different structural elements, i.e., two TMDs and an intervening soluble loop sequence. The relative contribution of these elements likely mediates the various aspects of p36 biogenesis including maintaining solubility before membrane insertion, targeting to mitochondria, and/or ensuring proper assembly in the mitochondrial outer membrane.

### The moderate hydrophobicity of TMD1 and TMD2 is essential for targeting p36 to mitochondria

**Figure 5:**
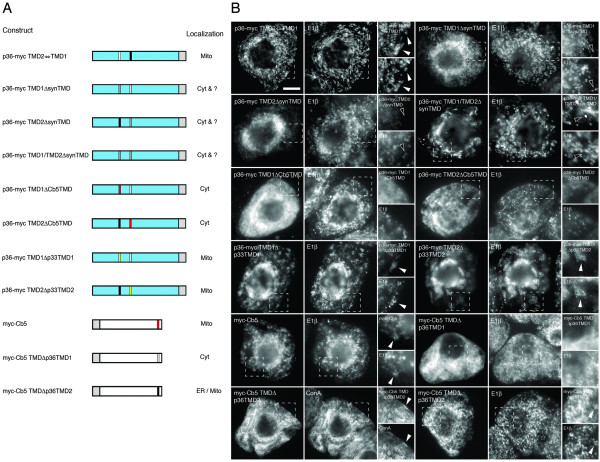
**Localization of p36 and Cb5 proteins with modified TMDs in BY-2 cells**. **(A) **Schematic illustrations of various p36-myc and myc-Cb5 proteins with modified TMDs and their corresponding subcellular localizations in transformed (via biolistic bombardment) BY-2 cells. The name of each mutant construct denotes p36-myc or myc-Cb5 and the specific alterations to its TMD(s). Hydrophilic portions of the p36 ORF are colorized blue, with TMD1 and TMD2 in p36 colored black and white, respectively. The single TMD in Cb5 is colorized red. Grey boxes denote the position of the myc epitope. Cyt, cytosol; ER, endoplasmic reticulum; Mito, mitochondria; ?, unknown subcellular compartment(s). **(B) **Representative (immuno)epifluorescence micrographs illustrating the localizations of the various constructs shown in (A). Each micrograph is labelled at the top left with the name of either the transiently-expressed protein, the endogenous mitochondrial marker protein, E1β, the endogenous peroxisomal marker protein, catalase, or ConA (fluor-conjugated Concanavalin A) serving as stain for the ER [89,98]. Hatched boxes represent the portion of the cells shown at higher magnification in the panels to the right. Solid arrowheads indicate obvious colocalizations; open arrowheads indicate obvious non-colocalizations. Bar = 10 μm.

The results presented above indicate that the sorting of p36 to mitochondria is, at least in part, dependent upon its TMDs. Thus, to test whether specific amino acid sequences or more global properties, e.g., overall hydrophobicity and/or length, within one or both of the TMDs contribute important mitochondrial targeting information, we generated several different mutant versions of p36-myc containing altered TMD sequences (Figure 5A). Included among these p36 TMD mutants were those that contained sequences derived from either TBSV p33 or the mitochondrial outer membrane-localized isoform of cytochrome *b*_5 _(Cb5), a tail-anchored protein whose mitochondrial targeting signal consists of several unique physicochemical and sequence-specific characteristics within its single C-terminal TMD and hydrophilic tail domain [27]. By replacing TMD1 or TMD2 in p36 with either the corresponding TMDs from p33 or the TMD from Cb5 (and vice versa, i.e., Cb5 with its TMD exchanged for TMD1 or TMD2 from p36), we sought to determine what role the p36 and p33 TMDs play in mitochondrial versus peroxisomal targeting and, in the case of p36 and Cb5, whether these two proteins possess functionally equivalent mitochondrial targeting information within their TMDs.

As shown in Figure 5B, when the sequences for TMD1 and TMD2 in p36 were exchanged the resulting mutant protein (p36-myc TMD1 ⇔ TMD2) localized exclusively to mitochondria. Interestingly, the morphology and distribution of the mitochondria in these cells, unlike in wild-type p36-myc-transformed cells (refer to Figure 1), did not appear to be altered (e.g., aggregated). These data suggest that the relative positions of the two TMDs within p36 influence the effects that this protein has on mitochondria morphology/distribution, but not its intracellular sorting.

An examination of the TMD amino acid sequences within p36 (underlined, Figure 4A) did not reveal any noticeable conserved features. For instance, with the exception that both TMDs possess similar (moderate) average hydrophobicity indexes (according to the Kyte-Doolittle algorithm [28]) of 1.58 (TMD1) and 2.05 (TMD2), they vary in their overall length (i.e., TMD1 is predicted to be 18 amino acid residues long, whereas TMD2 is 23 residues long). Moreover, they contain no obvious conserved amino acid sequence-specific motifs or an enrichment of particular amino acid residues. Therefore, the possibility was tested that the overall hydrophobic nature of the TMDs was the functional determinant in sorting p36 to mitochondria. Toward this end, either TMD1 or TMD2, or both TMDs together, in p36 were replaced with artificial (idealized) amino sequences composed of multiple -LALV-repeats [29] while still maintaining the length of the TMDs in p36, i.e., 18- and 23-long repeats of -LALV-corresponding to the length of TMD1 and TMD2 in p36, respectively. As shown in Figure 5B, all three of the resulting mutant versions of p36-myc (i.e., p36-myc TMD1ΔsynTMD, p36-myc TMD2ΔsynTMD and p36-myc TMD1/TMD2ΔsynTMD) mislocalized to cytosol and to some unknown punctate compartment(s) that did not colocalize with endogenous mitochondrial E1β (refer to open arrowheads in high magnified views shown in Figure 5B), nor peroxisomal catalase (data not shown). Similarly, replacement of TMD1 or TMD2 in p36 with the 18 amino-acid long single TMD from the mitochondrial isoform of Cb5 [27] resulted in both mutant proteins (p36-myc TMD1ΔCb5TMD and p36-myc TMD2ΔCb5TMD) being mislocalized to the cytosol. By contrast, both p36-myc TMD1Δp33TMD1 and p36-myc TMD2Δp33TMD2 in which TMD1 or TMD2 of p36 were replaced with TMD1 or TMD2 from p33, respectively, sorted to (aggregated) mitochondria in a manner similar to wild-type p36-myc (Figure 5B).

As shown also in Figure 5B, N-terminal myc-tagged Cb5 (myc-Cb5) localized exclusively to mitochondria, as previously published [27]. Replacement of the single TMD in myc-Cb5 with TMD1 or TMD2 from p36, however, resulted in the corresponding mutant proteins being mislocalized either to the cytosol (myc-Cb5Δp36TMD1) or to the ER (myc-Cb5Δp36TMD2) (Figure 5B); although in a small proportion (~15%) of the cells transformed with myc-Cb5Δp36TMD2 the mutant protein targeted to mitochondria in a manner similar to that of wild-type myc-Cb5 (Figure 5B).

Together, the data presented in Figure 5 imply that not all hydrophobic sequences are sufficient to replace TMD1 or TMD2 in p36 in terms of mediating its proper targeting to mitochondria. However, a pertinent question based on these data is why the TMDs from p33, a peroxisomal membrane-localized protein [15,25,30,31], but not an idealized hydrophobic TMD, nor the TMD from an authentic mitochondrial outer membrane protein Cb5 [27], are capable of preserving the targeting of p36 to mitochondria? One possibility is that the relatively moderate overall hydrophobicity of the TMDs in p36 is an essential feature of the protein's mitochondrial targeting signal. Based on this premise, the mislocalization of p36 when one (or both) of its TMDs was replaced with either an artificial TMD(s) or the Cb5 TMD was due to the relatively high hydrophobic indexes of these introduced sequences, i.e., 3.33 and 3.36 for the 18 and 23 amino acid-long synthetic TMDs and 2.42 for the 18 amino acid-long Cb5 TMD, compared to those for p36 TMD1 (1.58) and TMD2 (2.05). By contrast, the mitochondrial targeting of p36 was not disrupted when its TMDs were replaced individually with the relatively moderate hydrophobic TMDs from p33, i.e., 2.01 and 1.98 for TMD1 and TMD2, respectively.

This scenario that the mitochondrial sorting of p36 is mediated, at least in part, by the moderate hydrophobic nature of its TMDs is in agreement with results from several other studies on the sorting of authentic mitochondrial outer membrane proteins. That is, depending on the protein, a moderate hydrophobic TMD(s), combined with positive charges at its flanking soluble regions, is essential for mitochondrial outer membrane targeting [reviewed in [32,33]]. For example, the 20-kD and 70-kD subunits of the TOM complex (TOM20 and TOM70) and TOM45 (a mitochondrial outer membrane protein of 45 kD) from yeast all contain moderately hydrophobic TMDs that are functionally interchangeable in terms of their targeting behaviour [34]. The mammalian (human) TOM20 and yeast mitochondrial outer membrane protein fission 1 (FIS1) also appear to rely on the moderate hydrophobicity of their TMDs, since increasing their overall hydrophobic score via mutagenesis resulted in both modified proteins being mislocalized [35,36]. Conversely, reducing the overall hydrophobicity of an artificial TMD within a yeast ER-localized reporter membrane protein by introducing one or more hydrophilic residues caused it to relocalize from the ER to mitochondria [37].

A notable extension of this model is that, for at least some mitochondrial outer membrane proteins, they appear to rely also on subtle sequence-specific features within their TMDs. For instance, both the yeast 5-kD and 7-kD TOM subunits (TOM5 and TOM7) possess a conserved proline residue near the center of their TMDs that is required for their efficient targeting to mitochondria [38,39]. While the precise role of this proline in the mitochondrial targeting of these proteins has not been determined, it may be due to the residue's ability to destabilize the TMD's α-helical structure and, thus, dictate its overall configuration and subsequent interaction with the proper cognate receptor and/or integration/assembly factor(s) [32]. Interestingly, targeting of mitochondrial plant Cb5 also appears to rely on a conserved proline residue that forms part of a short hydrophilic surface situated within the protein's TMD [27]. Based on this, it is perhaps not surprising that replacement of the Cb5 TMD with either TMD1 or TMD2 from p36 resulted in both of the modified Cb5 proteins being either completely (myc-Cb5Δp36TMD1) or partially (myc-Cb5Δp36TMD2) mislocalized within cells (Figure 5B). That is, the nature of the targeting information within the TMDs of p36 and Cb5 appear to be sufficiently distinct in terms of their relative overall hydrophobicity and the presence (or the lack) of sequence-specific motifs, such that they are not functionally interchangeable. As discussed below, these apparent differences in the targeting elements within the TMDs of p36 and Cb5 also appear to reflect the relative contribution of unique positively-charged targeting elements at their flanking regions.

### The intervening loop sequence of p36 contains an amphipathic helix with a positively-charged face that is essential for mitochondrial targeting

**Figure 6:**
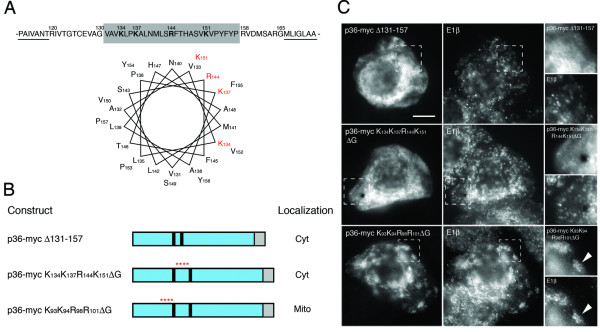
**Localization of p36 intervening-loop mutants in BY-2 cells**. **(A) **Deduced amino acid sequence of the intervening loop sequence (residues 120–165) and portions of the immediately-adjacent TMDs (underlined) in p36. The numbers shown above individual amino acids indicate their relative positions in p36. Amino acids predicted to form an α-helix (residues 131–157) are shaded grey and residues that are proposed to constitute the positively-charged face of the α-helix are bolded. Secondary structure prediction was carried out using Jpred . Numbers next to each amino acid residue shown in the α-helical wheel projection correspond to their relative positions in p36 (residues 121–157) beginning at the N-terminal end of the predicted α-helix. Positively-charged residues in the α-helix are colored red. **(B) **Schematic illustrations of various p36-myc mutant proteins and their corresponding subcellular localizations in transformed (via biolistic bombardment) tobacco BY-2 cells. The numbers in the name of each p36 mutant denote the specific amino acid residues that were either deleted or replaced with glycine residues. Portions of the p36 ORF are colorized blue or black, the latter denoting the two putative TMDs in p36. Asterisks highlight the relative position of the cluster of positively-charged amino acids that were replaced with glycine residues. Grey boxes denote the C-terminal myc epitope. Cyt, cytosol; Mito, mitochondria. **(C) **Representative (immuno)epifluorescence micrographs illustrating the localizations of the various constructs shown in (B). Each micrograph is labelled at the top left with the name of either the transiently-expressed protein or the endogenous mitochondrial marker protein, E1β. Hatched boxes represent the portion of the cells shown at higher magnification in the panels to the right. Arrowheads indicate obvious colocalizations. Bar = 10 μm.

Since the intervening loop sequence in p36 (residues 120–165 between TMD1 and TMD2) is significantly divergent from the corresponding loop sequence in peroxisomal-targeted p33 (Figure 4A) and since replacement of this region in p36 with that from p33 resulted in the hybrid protein (p33 103–131 p36-myc) being mislocalized to the cytosol (Figure 4B), we analyzed this region in p36 to determine if it contained any distinctive features involved in mitochondrial targeting. Figure 6A shows that the deduced amino acid sequence of the p36 intervening loop, specifically the central portion of this sequence that is devoid in p33 (residues 131–157, shaded in Figure 6A; refer also to Figure 4A), is enriched in both positively-charged (bolded) and hydroxylated (i.e., Ser, Thr and Pro) amino acid residues. This central region of the p36 intervening loop also has the propensity (based on secondary structure prediction programs) to form an α-helix, one with amphipathic characteristics conveyed by a positively-charged face (refer to amino acid residues in red; Figure 6A).

Based on these observations and previous reports that the targeting of several other authentic mitochondrial outer membrane-destined proteins rely on positively-charged residues together with a flanking TMD(s) [32,33], we examined further the positively-charged face of the amphipathic helix in p36 by mutational analyses. As shown in Figures 6B and 6C, deletion of amino acid residues 131–157 in p36-myc resulted in the mutant (i.e., p36-mycΔ131-157) being mislocalized to the cytosol. These data are consistent with the mislocalization of p36-myc when its intervening loop sequence was replaced with that of p33 (i.e., p33 103–131 p36-myc; Figure 4B).

Mitochondrial targeting of p36-myc was also abolished when glycine residues were exchanged for each of the four positively-charged residues predicted to be situated along the same face of the amphipathic α-helix within the protein's intervening loop sequence, i.e., p36-myc K_134_K_137_R_144_K_151_ΔG mislocalized to the cytosol (Figure 6C). By contrast, glycine substitutions of another cluster of positively-charged residues (i.e., K_93_K_94_R_98_R_101_) located immediately upstream of TMD1 did not disrupt the sorting of p36-myc to mitochondria (p36-myc K_93_K_94_R_98_R_101_ΔG; Figure 6C). These latter results, as well as those presented earlier for the mislocalization of p36 1-120-CAT (Figure 3) and p36 1–120 p33-myc (Figure 4) reinforce our conclusion that, in contrast to the findings reported by Weber-Lotfi et al. [18], the N-terminal hydrophilic region located upstream of TMD1 in p36 does not contain mitochondrial targeting information.

The results for p36-myc K_93_K_94_R_98_R_101_ΔG also indicate that not all mutations to the protein affected its mitochondrial targeting fidelity. An observation that underscores a substantive caveat of this study (and any analysis of membrane protein targeting) whereby mutations of p36 that resulted in the protein's mislocalization could have been due to aberrant protein folding, rather than a disruption of a specific targeting determinant(s). Although this possibility cannot be excluded, our combined use of loss-of-function and gain-of-function targeting assays strongly supports our definition of the *bona fide *mitochondrial targeting information within p36.

Collectively, our findings on the combined importance of positively-charged residues (Figure 6) and moderate hydrophobic TMDs (Figure 5) in the mitochondrial targeting of p36, as well as the N_out_-C_out _topological orientation of the protein in the mitochondrial outer membrane (Figure 2), suggests that the insertion and assembly of p36 involves a signal loop-anchor mechanism [40]. That is, interaction of the positively-charged residues in the intervening loop sequence of nascent p36 with the mitochondrial outer surface might promote a conformational change that drives insertion of its moderate hydrophobic TMDs into the bilayer, and results in the proper N_out_-C_out _topology. This proposed signal loop-anchor mechanism is consistent, as mentioned above, with results from previous *in vitro *insertion experiments with p36 and isolated mitochondria [18]. Moreover, since the topological orientation of p36 is identical to the topology of the authentic outer mitochondrial membrane proteins yeast fuzzy onion 1 (Fzo1) and its mammalian homologs, mitofusin (Mfn) 1 and 2, i.e., anchored in an N_out_-C_out _manner by two α-helical TMDs connected via a short loop sequence located in the intermembrane space [41,42], it is possible that the signal loop-anchor insertion of p36 is mediated by the same mitochondrial machinery that is utilized by Fzo1 and Mfn1/2. Results of experiments aimed at addressing this possibility are described next.

### p36 interacts with certain components of the mitochondrial TOM complex, but not with the mitochondrial SAM complex

Almost all mitochondrial proteins are encoded by nuclear genes, synthesized on free polyribosomes in the cytosol, and targeted post-translationally to the organelle. Thereafter, the recognition, translocation, and sorting of these proteins is usually mediated by the TOM complex and, depending on the protein's submitochondrial destination, one of several other translocase complexes located in the outer and inner mitochondrial membranes [reviewed in [43-45]]. For instance, based on studies carried out primarily with yeast and mammalian model systems, it is well established that nascent proteins destined for the mitochondrial matrix or inner membrane are recognized by the import receptors TOM20 and TOM70 and then delivered to one of two discrete translocase of the inner mitochondrial membrane (TIM) complexes. The majority of proteins destined for the outer mitochondrial membrane in yeast and mammals also rely on the TOM complex, but appear to do so in a number of different ways depending on their topology [reviewed in [33]]. For example, mitochondrial outer membrane proteins with multiple α-helical TMDs, including Mfn1/2, utilize the receptor TOM70, but not other components of the TOM complex [46]. β-barrel outer mitochondrial membrane proteins, such as porin and the channel forming subunit of the TOM complex, TOM40, as well as C-terminal tail-anchored subunits of the TOM complex (i.e., TOM22, TOM5, TOM6 and TOM7), are recognized mainly by TOM20 and then subsequently transferred to the SAM complex [47], a hetero-oligomeric complex located in the mitochondrial outer membrane where it functions in β-barrel protein insertion and assembly [33,48]. Interestingly, another subset of C-terminal, tail-anchored outer membrane proteins, including BAX and FIS1, do not require any known import (TOM) components, but instead appear to rely on the unique lipid composition of the mitochondrial outer membrane for their proper targeting and insertion [49,50].

While it is generally accepted that the various mitochondrial import pathways and associated machinery are conserved among evolutionarily diverse organisms including yeast, animals and plants, considerable differences exist among some of their core protein subunit compositions and functional specificities, particularly in plants [51,52]. Thus, p36 may or may not use the same mitochondrial insertion machinery in plants as that employed by multi-spanning mitochondrial outer membrane proteins in yeasts and mammals, such as the TOM70-dependent targeting of Mfn1/2 [46]. The targeting and insertion of p36 may be also more similar to that of yeast FIS1, which is mediated primarily by the distinct lipid composition of the mitochondrial outer membrane [50], a possibility that appears to be consistent with previous data indicating that p36 does not to rely on any mitochondrial-surface proteinaceous receptors [18]. This latter conclusion, however, was based solely on the observation that pre-treatment of isolated mitochondria with trypsin to remove any exposed parts of surface receptors did not prevent membrane insertion of p36. Indeed, this apparent lack of sensitivity to trypsin is not evidence *a priori *that p36 import does not utilize a proteinaceous receptor(s), since mitochondrial surface receptors can display differences in terms of their sensitivity/resistance to an applied protease [53,54] and can even be bypassed *in vitro *such that a chloroplast protein can be imported into protease-pretreated mitochondria with the same efficiency as a mitochondrial protein that normally relies on a surface receptor [55].

To determine the insertion pathway of p36 into the outer mitochondrial membrane we tested whether it could interact directly with a variety of protein components of the plant TOM and/or SAM complexes. Specifically, we employed bimolecular fluorescence complementation (BiFC) assays to determine whether *in vivo *physiological interactions occur between p36 and four *Arabidopsis *TOM protein subunits including: i) the TOM20 import receptor [56-58]; ii) TOM22, which may function in a manner similar to yeast and mammalian TOM22 by providing some receptor function, but mostly as a link between the primary receptors TOM20 and TOM70 [59]; iii) TOM40, the central pore-forming subunit of the TOM complex [56]; and iv) mtOM64 (outer mitochondrial membrane protein of 64-kD), which functions as an import receptor and, based on structural similarities, may be a paralog of yeast and mammalian TOM70 [52,60]. We also tested whether p36 physically interacts with *Arabidopsis *METAXIN, a diverged form of the SAM component SAM37 and METAXIN1 in yeasts and mammals, respectively [52,61].

**Figure 7:**
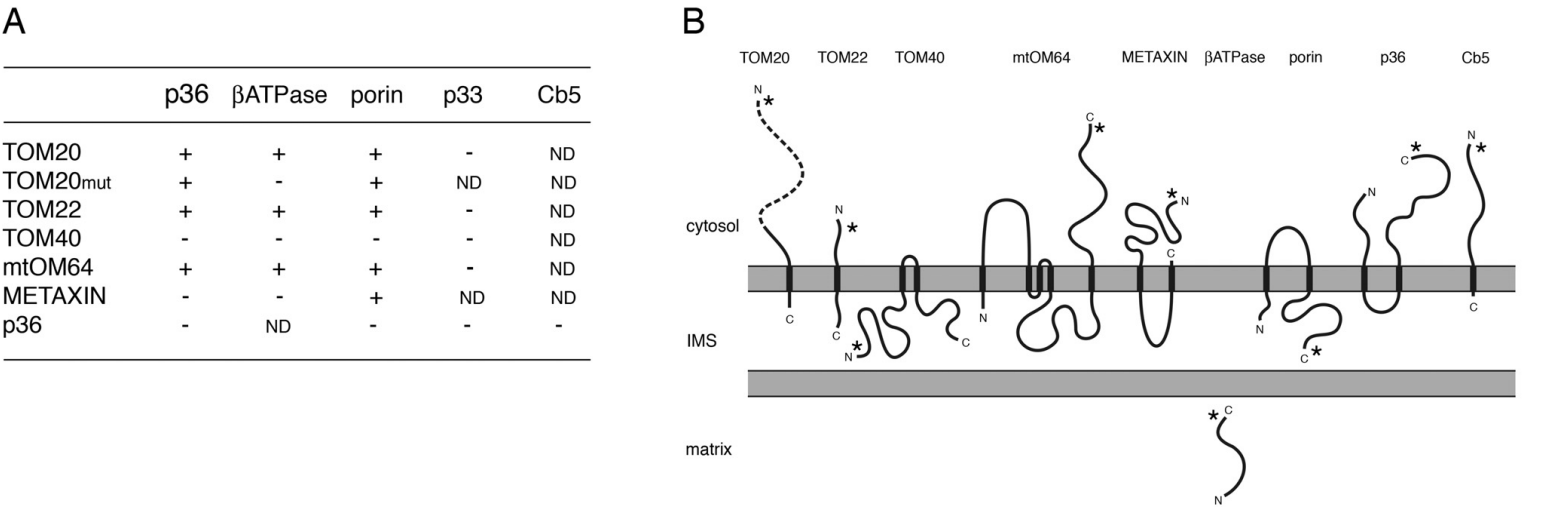
**BiFC analysis of interactions among p36 and components of the TOM and SAM complexes**. **(A) **BY-2 cells were co-transformed (via biolistic bombardment) with either p36, the N-terminal mitochondrial matrix targeting presequence of the β subunit of the F_1_-ATPase (βATPase), porin, p33, or Cb5 fused at either their N or C termini to cVenus (columns) and various constituents of the TOM complex (or an N-terminal mutant version of TOM20; TOM20mut), METAXIN, or p36 fused at either their N or C termini to nVenus (rows). In addition, all cells were co-transformed with either RFP, which served as a convenient means of identifying transformed cells. At ~16 h post-bombardment, cells were formaldehyde fixed and viewed by epifluorescence microscopy. Interactions were scored based on either the presence (+) or absence (-) of a BiFC (Venus) signal relative to reconstitution controls in which individual Venus half fusion proteins were co-expressed with the corresponding "empty" vector containing nVenus or cVenus fragments alone. All BiFC signals observed co-localized with (co)expressed mitochondrial βATPase-GFP. For each pair of plasmids tested, > 25 transformed cells were scored from at least two independent transformation (biolistic bombardment) experiments. ND, not determined. **(B) **Predicted topologies of mitochondrial outer membrane proteins used in BiFC experiments, the results of which are presented in (A). Also shown is the localization of the soluble βATPase N-terminal mitochondrial targeting presequence in the matrix following import. The topological orientations of mitochondrial outer membrane proteins shown are based on differential permeablization results presented either in Figure 2 (for p36), in Additional file 3A (for TOM proteins, METAXIN, and porin) or published previously in Hwang et al. [27] (for Cb5). Regions of proteins proposed to be hydrophobic membrane-spanning domains or hydrophilic domains facing the cytosol, intermembrane space or matrix were identified using the TMpred program . The dashed line in the N-terminal portion of TOM20 represents the region of the receptor that was deleted in the corresponding mutant (TOM20_mut_); specifically, protein's N-terminal 1–142 residues including the tetratricopeptide motif-based receptor domain for mitochondrial precursor proteins [58]. Asterisks indicate the relative position of nVenus or cVenus and the immediately adjacent myc- or HA-epitope tag appended to each protein. IMS, intermembrane space.

BY-2 cells were transiently transformed with pairs of plasmids encoding p36 fused at its C terminus to the C-terminal half of the modified yellow fluorescent protein Venus [62] (cVenus) and one of the TOM or SAM proteins fused at either their N or C termini to the N-terminal half of Venus (nVenus) (e.g., nVenus-TOM20); refer to illustrations of various proteins in Figure 7B showing their predicted topological orientations in the mitochondrial outer membrane and the relative location of their appended Venus fragment. Because each half of Venus is not intrinsically fluorescent, Venus fluorescence is observed only when intermolecular interactions occur between nVenus- and cVenus-tagged proteins [63-65], highlighting an advantage of BiFC over other protein-protein interaction assays (e.g., yeast two-hybrid and immunoprecipitations) in that the subcellular localization of the interacting proteins can directly be observed in living cells via microscopy. All cells were also co-bombarded with a third plasmid encoding the red fluorescent protein (RFP), which served as an internal standard for transformation efficiency and aided in identifying transformed cells.

Alternatively, BY-2 cells were co-bombarded with a plasmid coding for an individual nVenus- or cVenus-tagged protein along with a plasmid encoding βATPase-GFP (consisting of the N-terminal mitochondrial matrix targeting presequence of βATPase [residues 1–60] fused to the N terminus of GFP), which served to identify transformed cells and, acting as a well-characterized mitochondrial marker protein [66,67]. As presented in Additional file 3A, results from these latter experiments provided confirmation of the expression of each of the individual Venus half fusion proteins (via immunomicroscopy of an appended myc or hemaglutinin (HA)-epitope tag; see Methods and Materials for details on the construction of BiFC plasmids), as well as their mitochondrial localization. Additional results confirming the topological orientation of several of these Venus half fusion proteins in BY-2 cells using the differential detergent permeabilization assay are presented in Additional file 3B.

The results presented in Figure 7A show that p36 (i.e., p36-cVenus) interacted *in vivo *with all three of the nVenus-tagged versions of the putative import receptors, TOM20, TOM22 and mtOM64. Notably, the interaction of p36 with TOM20 was unaffected when the receptor protein's N-terminal tetratricopeptide motif-based domain responsible for the recognition and interaction with mitochondrial precursor proteins (residues 1–142; [58]) was deleted (TOM20mut), suggesting that the interaction of p36 with the TOM20 is not mediated by this substrate-receptor-binding motif. p36 did not interact, however, with either TOM40, the SAM component METAXIN, or itself (i.e., p36-nVenus) (Figure 7A). While these latter data appear to contradict those published elsewhere for the homo-oligmerization of the p36-related protein, TBSV p33 [[68] and references therein] and, p36-p36 and other p36-protein interactions likely occur within the context of the functional CIRV replication complex and, thus, mediated by the allied replication protein, p95, as well viral RNA template [69].

In a series of control experiments we also evaluated whether or not the βATPase matrix targeting presequence and/or the full-length outer mitochondrial β-barrel membrane protein, porin, interacted with the TOM and SAM complex protein subunits in a manner similar to p36. As shown in Figure 7A, the data obtained for βATPase and porin are mostly in accordance with the current working models for the mitochondrial import pathways used by these two proteins [43-45,51]. For instance, both βATPase and porin (βATPase-cVenus and porin-cVenus) interacted with the nVenus-tagged versions of TOM20, TOM22 and mtOM64, reflecting the apparent functional redundancy of these import receptors in plants [52]. Likewise, porin interacted with the N-terminal mutant version of TOM20 (TOM20mut), but βATPase did not, supporting the notion that the import of matrix-destined proteins is dependent primarily on TOM20 via the receptor's N-terminal tetratricopeptide motif domain [56,58]. The relative importance of the mtOM64 receptor in βATPase and porin (and p36) import using a similar mutagenesis-BiFC approach was not, however, assessed, since deletion of the predicted substrate-receptor binding motif in mtOM64 [60,70] disrupted its sorting to mitochondria in BY-2 cells (data not shown).

Figure 7A shows also that porin, but not βATPase, interacted with METAXIN, consistent with the proposed role of this SAM component in the insertion and assembly of β-barrel proteins in yeasts, mammals and plants [47,48,52]. However, Lister et al. [52] recently showed using pull-down and yeast two-hybrid assays that METAXIN interacted with a number of plant matrix-destined precursor proteins as well as β-barrel porin and TOM40, suggesting that this protein receptor, unlike its counterparts in yeast and mammals, performs a variety of roles in plants in addition to the insertion and assembly of β-barrel membrane proteins. Other than the use of different experimental approaches in the latter study [52] compared to that employed here, a reason(s) for the apparent lack of interaction between βATPase and METAXIN is not known. Regardless, these results for βATPase (and p36) and METAXIN, combined with the negative interaction data for certain other protein pairs discussed below (e.g., p36 and Cb5) provides convincing evidence that the Venus fluorescence observed in (positive) BiFC assays (e.g., porin and METAXIN) resulted from specific protein-protein interactions and was not simply a consequence, for example, of the overexpression of protein pairs localized to the same intracellular local (i.e., mitochondrial outer membrane) [65].

At least one unexpected finding from the BiFC experiments presented in Figure 7A was that both βATPase and porin (and p36) did not interact with TOM40, the putative primary subunit of the TOM complex import channel [56]. These results contradict those obtained primarily from studies with yeasts and mammals, wherein TOM40 participates in the import of most types of mitochondrial proteins including those destined for the matrix or outer membrane [43-45]. One possible reason for this apparent lack of interaction between TOM40 and βATPase and porin (and p36) is that this putative channel protein, while being efficiently targeted to mitochondria when expressed individually in BY-2 cells (see Additional file 3A), was not properly inserted/assembled into functional TOM complexes and, thus, could not participate in mitochondrial protein import. Another possible reason may be that the nVenus fragment appended to TOM40 (refer to illustration in Figure 7B) is sterically inaccessible, such that even if the fusion protein was properly assembled into the TOM complex it could not interact with cVenus appended to either co-expressed βATPase, porin or p36. Unfortunately, since another TOM40 fusion protein with nVenus appended to its C terminus (TOM40-nVenus) was not sorted to mitochondria in BY-2 cells (data not shown) and because no other experimental data has been published for the structure and/or function of a plant TOM40, resolution of this issue remains to be addressed.

In additional control experiments TBSV p33 (p33-cVenus) was not observed to interact with any of the TOM complex subunit proteins tested nor with p36 (Figure 7A). These results were entirely expected since p33 sorts to peroxisomes and not to mitochondria ([25]; see Additional Figure 3A for the localization of p33-cVenus in BY-2 cells). Interactions were also not observed between p36 and porin or p36 and Cb5 (Figure 7A), indicating that, similar to the negative results discussed above for βATPase (and p36) and METAXIN, the positive Venus signals detected for certain other protein pairs was due to their specific protein-protein interaction. However, related to these control experiments with p36, porin and Cb5, it is important to note that, whereas Cb5 and p36 are orientated in outer mitochondrial membranes in an N_out_-C_in _[27] and N_out_-C_out _manner (Figure 2; and [15,18]), respectively, porin contains two putative TMDs and orientated in an N_in_-C_in _manner (Figure 7B; refer also to differential permeabilization results for cVenus-porin in Additional file 3B). Thus, while cVenus appended to porin (porin-cVenus) is predicted to be located in the intermembrane space, nVenus appended to p36 (p36-nVenus) faces the cytosol (Figure 7B) and, thus, the lack of a BiFC signal for these two proteins when co-expressed in BY-2 cells was expected given the physical separation of their corresponding Venus halves [65].

Taken together, the BiFC results presented in Figure 7A indicate that p36 interacts with a variety of import receptors in the TOM complex including TOM20, TOM22, and mtOM64, consistent with recent findings that, compared to yeasts and mammals, the mitochondrial import apparatus in plants is highly flexible with overlapping specificity [52]. Our BiFC results indicate also that whereas the porin interacts with the putative SAM component METAXIN, p36 (and βATPase) does not. Hence, METAXIN appears to participate specifically in the assembly of outer mitochondrial membrane proteins containing a β-barrel structure, a conclusion that extends the findings presented previously for the role of this mitochondrial import component in plants [52].

## Conclusion

Viruses are well known for their ability to exploit specific organelles and pathways in infected host cells in order to facilitate their replication [1-3]. Here we showed that p36, an essential component of the CIRV replication complex and a key factor involved in the virus' ability to transmogrify mitochondria into MVBs in infected plant cells, contains a prototypic mitochondrial outer membrane targeting signal. That is, similar to the targeting signals characterized for most authentic mitochondrial outer membrane proteins in evolutionarily diverse organisms [32,33], the p36 targeting signal consists of a cluster of positively-charged residues flanked by moderately hydrophobic TMDs. We showed also that p36 interacts with several TOM components, specifically the import receptors TOM20, TOM22 and mtOM64. These results, combined with the apparent conservation of a functional outer mitochondrial targeting signal in p36, indicate that CIRV has evolved a strategy to utilize the host-cell mitochondrial sorting pathways in order to control or modulate the biogenesis and/or function of the organelle to its own advantage. For instance, mitochondrial-derived MVBs in CIRV-infected cells could provide the necessary structural framework or platform for replication complex assembly and/or provide a sophisticated protective structure that shields the nascent viral RNA or viral replication proteins from degradation. Both of these possibilities have been proposed previously for other viruses that also rely on the mitochondrial outer membrane for their replication [71-75].

While the functional significance of the CIRV-mitochondria relationship and the molecular mechanisms underlying the membrane rearrangements that occur during mitochondrial MVB biogenesis in CIRV-infected cells remain to be elucidated, future studies aimed at understanding these processes, similar to results presented here for the sorting of p36, will undoubtedly provide insights to certain aspects of normal mitochondrial biogenesis in plant cells (e.g., membrane remodelling during mitochondria fission). To date, only one plant outer mitochondrial membrane protein, Cb5, has been well examined in terms of its targeting signal and insertion mechanism [27,76-78]. Thus, as other plant outer mitochondrial membrane proteins begin to be investigated, including those identified using proteomic- and/or bioinformatics-based approaches [79-83], the properties of the p36 targeting signal and its import mechanism will serve as a useful reference.

## Methods

### Recombinant DNA procedures and reagents

Standard recombinant DNA procedures were performed as described by Sambrook et al. [84]. Molecular biology reagents were purchased either from New England BioLabs (Beverly, MA, USA), Promega (Madison, WI, USA), Perkin-Elmer (Perkin-Elmer Biosystems, Mississauga, Canada), Stratagene (La Jolla, CA, USA), or Invitrogen (Carlsbad, CA, USA). Synthetic oligonucleotides were synthesized by either Invitrogen (Frederick, MD) or University of Guelph Laboratory Services (Guelph, Canada); a complete list of the sequences of the oligonucleotides primer used in plasmid constructions described below are provided in Additional file 4. Plasmid DNA was isolated using Qiagen reagents (Qiagen, Mississauga, Canada), and dye terminated cycle sequencing was used with an Applied Biosystems Model 377 or 3730 automated sequencer (Perkin-Elmer Biosystems) to verify all DNA constructs. Mutagenesis was carried out using appropriate complementary forward and reverse mutagenic primers and procedures according to the QuickChange site-directed mutagenesis kit (Stratagene).

### Construction of plasmids

#### Plasmids containing p36 and/or p95

pRTL2/p36, encoding the CIRV 36 kDa replicase protein was constructed in the following manner. First, sequences encoding the p36 ORF were amplified via the polymerase chain reaction (PCR) (primers Fp135 and Rp107; Additional file 4) and the plasmid pUC18/CIRV (purchased from the American Type Culture Collection, clone PVMC-47) serving as template DNA. This PCR also introduced a 5' *Nco*I site and a 3' *Xma*I site that replaced the p36 stop codon. The resulting PCR products were ligated into pCR2.1 TOPO (Invitrogen) followed by ligation of the *Nco*I-*Xma*I fragment from the resulting plasmid (pCR2.1 TOPO/p36-*Xma*I) into *Nco*I-*Xma*I-digested pRTL2, a plant expression vector containing the Cauliflower Mosaic Virus (CaMV) 35S promoter and terminator and the tobacco etch potyvirus leader sequence for enhanced translation [85]. Next, a stop codon was introduced via PCR site-directed mutagenesis (primers Fp447 and Rp448) at the 3' end of the p36 ORF in pRTL2/p36-*Xma*I, yielding pRTL2/p36.

The C-terminal myc-epitope-tagged version of p36 in pRTL2 (pRTL2/p36-myc) was constructed by annealing complementary oligonucleotides (Fp177 and Rp178) that encoded the myc-epitope tag (-EQKLISEEDL-; [22]), a stop codon and *Xma*I overhangs, then ligating these into *Xma*I-digested pRTL2/p36-*Xma*I. An N-terminal myc-epitope-tagged version of p36 (pRTL2/myc-p36) was constructed by annealing complementary oligonucleotides (Fp29 and Rp30) encoding a start codon, the myc epitope and *Nco*I overhangs, and then ligating these into *Nco*I-digested pRTL2/p36.

To construct pRTL2/Rep, sequences encoding both CIRV replicase proteins (p36 and p95) were amplified via PCR (primers Fp135 and Rp572) from pUC18/CIRV with 5' *Nco*I and 3' *Xma*I sites. The resulting PCR products were digested with *Nco*I and *Xma*I and ligated into *Nco*I-*Xma*I-digested pRTL2, yielding pRTL2/Rep. pRTL2/p95 was generated by mutating via site-directed mutagenesis (primers Fp642 and Rp641) the p36 amber stop codon at nucleotide positions 1067–1069 to a tyrosine.

An infectious cDNA of CIRV, whereby the sequence for the entire CIRV genome [8] was cloned between a 5' CaMV 35S promoter and 3' hepatitis delta virus ribozyme, was constructed in three steps. First, a unique *Stu*I site was introduced via site-directed mutagenesis (primers Fp646 and Rp645) upstream of the TBSV sequence in the plasmid pHST20 [86], yielding pHST20/*Stu*I-*Xma*I. Digestion of this plasmid with *Stu*I and *Xma*I allowed for the removal of sequences coding for the entire TBSV genome. Second, sequences consisting of the first 621 nucleotides of the CIRV genome were amplified via PCR (primers Fp735 and Rp734 and pUC18/CIRV as template DNA) along with a 5' *Stu*I site. The resulting PCR products were then digested with *Stu*I and *Sph*I by taking advantage of a unique *Sph*I site within the amplified region of the CIRV genome. Third, the remaining sequences for the CIRV genome (nucleotides 622 to 4760) were excised from pUC18/CIRV with *Sph*I and *Xma*I and then this fragment and the *Stu*I-*Sph*I fragment were ligated together into *Stu*I/*Xma*I-digested pHST20/*Stu*I-*Xma*I, yielding pHST/CIRV.

#### Plasmids containing p36-CAT or p36-GFP fusion constructs

Construction of p36-CAT fusion constructs was carried out by first introducing various silent restriction sites at specific localizations within the p36 ORF. For instance, a silent *Mlu*I site was introduced via PCR site-directed mutagenesis (primers Fp275 and Rp276) at the 3' end of the first TMD in the p36 ORF (amino acid residues 119 and 120) to yield pRTL2/p36-myc (*Mlu*I). In a second mutagenesis reaction (primers Fp277 and Rp278) a silent *Avr*II site was introduced immediately after the second TMD in the p36 ORF (amino acid residues 189 and 190) in the plasmid pRTL2/p36-myc (*Mlu*I), yielding pRTL2/p36-myc (*Mlu*I/*Avr*II).

To construct pRTL2/p36-CAT, encoding the entire p36 ORF fused to the N terminus of CAT, the *Xma*I site in pRTL2/p36-*Xma*I was replaced via site-directed mutagenesis (primers Fp340 and Rp341) with an *Avr*II site to yield pRTL2/p36-*Avr*II. Next, the p36 ORF was excised from pRTL2/p36-*Avr*II with *Nco*I and *Avr*II and ligated into *Nco*I/*Avr*II-digested pRTL2/*Nco*I/*Mlu*I/*Avr*II-CAT. pRTL2/*Nco*I/*Mlu*I/*Avr*II-CAT is a general purpose cassette vector containing the CAT ORF (minus a start codon) and a convenient 5' multiple cloning site (MCS) including in-frame *Nco*I, *Mlu*I and *Avr*II sites [25]. pRTL2/p36 1-190-CAT, encoding the N-terminal 190 residues from p36 fused to the N-terminus of CAT, was generated by digesting pRTL2/p36-myc (*Mlu*I/*Avr*II) with *Nco*I and *Avr*II and ligating the resulting fragment into *Nco*I-*Avr*II-digested pRTL2/*Nco*I/*Mlu*I/*Avr*II-CAT. To generate pRTL2/p36 1-120-CAT, the CAT ORF was excised from pRTL2/*Mlu*I/*Avr*II-CAT (a modified version of pRTL2/*Nco*I/*Mlu*I/*Avr*II-CAT lacking the unique *Nco*I site in the MCS; [25]) with *Mlu*I and *Xba*I (located immediately 3' of the CAT ORF) and the resulting fragment was ligated into *Mlu*I/*Xba*I-digested pRTL2/p36-myc (*Mlu*I/*Avr*II). pRTL2/p36 120-190-CAT was constructed by first replacing via site-directed mutagenesis (primers Fp331 and Rp332) the *Mlu*I site in pRTL2/p36-myc (*Mlu*I/*Avr*II) with *Nco*I to yield pRTL2/p36-myc (*Mlu*IΔ*Nco*I/*Avr*II). pRTL2/p36-myc (*Mlu*IΔ*Nco*I/*Avr*II) was then digested with *Nco*I and *Avr*II and the resulting fragment corresponding to a start codon and amino acids 120 to 190 in the p36 ORF was ligated into *Nco*I/*Avr*II-digested pRTL2/*Nco*I/*Mlu*I/*Avr*II-CAT. pRTL2/p36 120-190-CAT was then used a template DNA in a site-directed mutagenesis reaction (primers Fp407 and Rp408) to delete sequences encoding amino acids 166 to 190 in the p36 ORF and corresponding to the p36 TMD2, yielding pRTL2/p36 120-164-CAT. pRTL2/p36 90-164-CAT and pRTL2/p36 90-190-CAT were constructed by amplifying via PCR (primers Fp449 and Rp453, and Fp457 and Rp453-2, respectively), along with pRTL2/p36-myc (*Mlu*IΔ*Nco*I/*Avr*II) as template DNA, sequences coding for either amino acids 90 to 164 or 90 to 190 in the p36 ORF along with 5' *Nco*I and 3' *Avr*II sites. Next, the PCR products were digested with *Nco*I and *Avr*II and ligated into *Nco*I/*Avr*II-digested pRTL2/*Nco*I/*Mlu*I/*Avr*II-CAT.

#### Plasmids containing modified versions of p36 or p33

pRTL2/p33 1-156-p36-myc, pRTL2/p33 103-156-p36-myc and pRTL2/p33 103-131-p36-myc, consisting of p33 amino acids 1 to 156, 103 to 156, and 103 to 131, respectively, within the context of p36-myc, were constructed as follows. pRTL2/p33 1-156-p36-myc was constructed by digesting pRTL2/p33-myc (*Mlu*I) with *Nco*I and *Avr*II and ligating the resulting fragment into *Nco*I/*Avr*II-digested pRTL2/p36-myc (*Mlu*I/*Avr*II). pRTL2/p33-myc (*Mlu*I) is a modified version of pRTL2/p33-myc [25] in which codons 104 and 105 in the p33 ORF were replaced via PCR site-directed mutagenesis (primers Fp273 and Rp274) with a silent *Mlu*I site. pRTL2/p33 103-120-p36-myc was constructed by digesting pRTL2/p33-myc (*Mlu*I) with *Mlu*I and *Avr*II and ligating the resulting fragment into *Mlu*I/*Avr*II-digested pRTL2/p36-myc (*Mlu*I/*Avr*II). pRTL2/p33 103-131-p36-myc was constructed in two steps. First, annealed complementary oligonucleotides (Fp1983 and Rp1984) encoding amino acids 103–131 from p33 along with 5' *Mlu*I and 3' *Sal*I overhangs were ligated into *Mlu*I-*Sal*I-digested pRTL2/p36-myc (*Mlu*I/*Sal*I/*Avr*II), yielding pRTL2/p33 103-131-VDMSAR-p36-myc. Second, sequences encoding the remaining p36 amino acids -VDMSAR- at the 3' end of the loop sequence in pRTL2/p33 103-131-VDMSAR-p36-myc were removed using site-directed mutagenesis (primers Fp2007 and Rp2008). pRTL2/p36-myc (*Mlu*I/*Sal*I/*Avr*II) was generated by introducing a silent *Sal*I site via site-directed mutagenesis (primers Fp576 and Rp575) upstream of TMD2 in the p36 ORF in pRTL2/p36-myc (*Mlu*I/*Avr*II).

pRTL2/p36 1-190-p33-myc, pRTL2/p36 1-120-p33-myc, and pRTL2/p36 120-190-p33-myc, consisting of p36 amino acids 1 to 190, 1 to 120, and 120 to 190, respectively, within the context of p33-myc, were constructed as follows. pRTL2/p36 1-190-p33-myc was constructed by digesting pRTL2/p36-myc (*Mlu*I/*Avr*II) with *Nco*I and *Avr*II and ligating the resulting fragment into *Nco*I/*Avr*II-digested pRTL2/p33-myc (*Mlu*I). pRTL2/p36 1-120-p33-myc was constructed by digesting pRTL2/p36-myc (*Mlu*I/*Avr*II) with *Nco*I and *Mlu*I and ligating the fragment into *Nco*I/*Mlu*I-digested pRTL2/p33-myc (*Mlu*I). pRTL2/p36 120-190-p33-myc was constructed by digesting pRTL2/p36-myc (*Mlu*I/*Avr*II) with *Mlu*I and *Avr*II and ligating the fragment into *Mlu*I/*Avr*II-digested pRTL2/p33-myc (*Mlu*I).

Plasmids encoding modified versions of p36-myc in which TMD1 and TMD2 were either swapped or replaced (individually or together) with synthetic TMDs were generated as follows. pRTL2/p36-myc TMD1 ⇔ TMD2, in which TMD1 of p36 was replaced with TMD2 of p36 and vice versa, was constructed by first ligating annealed, complementary synthetic oligonucleotides (Fp654 and Rp653) coding for p36 TMD2 (amino acids 166–188) along with 5' *Sna*BI and 3' *Mlu*I overhangs into *Sna*BI/*Mlu*I-digested pRTL2/p36-myc (*Sna*BI/*Mlu*I/*Avr*II), yielding pRTL2/p36-myc TMD1ΔTMD2. pRTL2/p36-myc TMD1ΔTMD2 was then used as template DNA to introduce via site-directed mutagenesis (primers Fp576 and Rp575) a silent *Sal*I site upstream of TMD2 in the p36 ORF, yielding pRTL2/p36-myc TMD1ΔTMD2-*Sal*I. Next, two sets of synthetic complementary oligonucleotides (Fp682 and Rp680, and Fp681 and Rp679) coding for TMD1 of p36 along with 5'*Sal*I and 3' *Avr*II overhangs were annealed and ligated into *Sal*I/*Avr*II-digested pRTL2/p36-myc TMD1ΔTMD2-*Sal*I, yielding pRTL2/p36-myc TMD1 ⇔ TMD2.

pRTL2/p36-myc TMD1ΔsynTMD and pRTL2/p36-myc TMD2ΔsynTMD, in which TMD1 and TMD2 of p36, respectively, were replaced with an artificial/idealized TMD consisting of multiple -LALV-amino acid repeats [29], were constructed in the following manner. Synthetic complementary oligonucleotides coding for synthetic TMDs of the same length as predicted for p36 TMD1 (18 residues) or TMD2 (23 residues), along with either 5' *Sna*BI and 3' *Mlu*I overhangs (Fp1670 and Rp1672) or 5' *Sal*I and 3' *Avr*II overhangs (Fp1674 and Rp1675), were annealed and ligated into either *SnaB*I/*Mlu*I-digested pRTL2/p36-myc (*SnaB*I/*Mlu*I/*Avr*II) or *Sal*I/*Avr*II-digested pRTL2/p36-myc (*Mlu*I/*Sal*I/*Avr*II). To construct pRTL2/p36-myc TMD1/TMD2ΔsynTMD, in which both p36 TMDs were replaced with the artificial/idealized TMDs composed of -LALV-repeats, pRTL2/p36-myc TMD2ΔsynTMD was digested with *Xba*I and the resulting fragment ligated into *Xba*I-digested pRTL2/p36-myc TMD1ΔsynTMD.

pRTL2/p36-myc TMD1ΔCb5TMD was constructed by ligating annealed complementary oligonucleotides (Fp1977 and Rp1978) coding for the 18 amino-acid-long TMD of the tung mitochondrial isoform of Cb5 (isoform D) [27] with 5' *Sna*BI and 3' *Mlu*I overhangs into *Sna*BI-*Mlu*I-digested pRTL2/p36-myc (*Sna*BI/M*lu*I/*Avr*II). Similarly, pRTL2/p36-myc TMD2ΔCb5TMD was constructed by ligating annealed complementary oligonucleotides (Fp1979 and Rp1980) coding for the p36 amino acid sequence -VDMSARG-immediately adjacent (upstream) to TMD2 and the tung Cb5 TMD into *Sal*I-*Avr*II-digested pRTL2/p36-myc (*Mlu*I/*Sal*I/*Avr*II). pRTL2/myc-Cb5Δp36 TMD1 and pRTL2/myc-Cb5Δp36 TMD2 encoding an N-terminal myc-tagged version of tung mitochondrial Cb5 with its single C-terminal TMD replaced with either TMD1 or TMD2 from p36 were constructed by ligating annealed complementary oligonucleotides coding for either the p36 TMD1 (Fp1960 and Rp1961) or p36 TMD2 (Fp1962 and Rp1963) and the Cb5 C-terminal hydrophilic (tail) sequence -RKK-COOH, along with a stop codon and 5' *Nhe*I and 3' *Xba*I overhangs into *Nhe*I-*Xba*I-digested pRTL2/myc-Cb5. Details on the construction of pRTL2/myc-Cb5 have been published elsewhere [27].

pRTL2/p36-myc TMD1Δp33TMD was constructed by ligating annealed complementary oligonucleotides (Fp113 and Rp114) coding for the p33 TMD1 (residues 84–98) and the amino acid sequence -SYA-immediately downstream of TMD1 with 5' *Sna*BI and 3' *Mlu*I overhangs into *Sna*BI-*Mlu*I-digested pRTL2/p36-myc (*Sna*BI/M*lu*I/*Avr*II). Similarly, pRTL2/p36-myc TMD2Δp33TMD2 was constructed by ligating two sets of annealed complementary oligonucleotides (Fp130A and Rp131A and Fp130B and Rp130B) coding for the p33 TMD2 (residues 132–156) with 5' *Sal*I and 3' *Avr*II overhangs into *Sal*I-*Avr*II-digested pRTL2/p36-myc (*Mlu*I/*Sal*I/*Avr*II).

pRTL2/p36-myc Δ 131–157 coding for p36-myc lacking amino acids residues 131–157 within the intervening loop sequence was generated using site-directed mutagenesis (primers Fp1966 and Rp1967), along with pRLT2/p36-myc as template DNA. pRTL2/p36-myc K_93_K_94_R_98_R_101_ΔG in which the positively-charged amino acid residues at positions 93, 94, 98 and 101 in the p36 ORF were each replaced with a glycine were constructed using site-directed mutagenesis (primers Fp412 and Rp410), along with pRTL2/p36-myc as template DNA. pRTL2/p36-myc K_134_K_137_R_144_K_151_ΔG was constructed in two steps. First, sequences encoding arginine at position 144 and lysine at position 151 in p36-myc (pRTL2/p36-myc) were mutated to glycines using site-directed mutagenesis (primers Fp2048 and Rp2049), yielding pRTL2/p36-myc R_144_K_151_ΔG. Second, sequences encoding the lysines at positions 134 and 137 in pRTL2/p36-myc R_144_K_151_ΔG were mutated to glycines using site-directed mutagenesis (primers Fp2098 and Rp2099), yielding pRTL2/p36-myc K_134_K_137_R_144_K_151_ΔG.

#### Plasmids used for BiFC

BiFC plasmids used in this study contained fragments derived from Venus, a variant of the yellow fluorescent protein (YFP) [62], and were constructed by replacing sequences encoding the N- or C-terminal fragments from YFP in the pSATN BiFC series of vectors [63] with sequences encoding the corresponding Venus N-terminal (nVenus) or C-terminal (cVenus) fragments. Venus was selected as the fluorescent protein for these redesigned BiFC plasmids because, compared to YFP, it does not require preincubation of cells at lower temperatures to facilitate efficient chromophore maturation [87]. Moreover, Venus is brighter than YFP, thus requiring lower amounts of plasmid(s) for transformations and a shorter incubation time, features that increase both the BiFC signal and specificity in terms of protein-protein interactions [64]. Overall, four different pSATN-based Venus BiFC vectors containing either nVenus or cVenus, an adjacent myc- or HA-epitope tag [22] serving as convenient means to immunodistinguish each (co-)expressed fusion protein, and a 5' or 3' MCS for cloning of the test gene were generated, including: pSAT4/nVenus C1, pSAT4/nVenus N1, pSAT4/cVenus C1 and pSAT4/cVenus N1. Details on the construction of these plasmids are as follows.

To construct pSAT4/nVenus C1, sequences encoding the N-terminal 174 amino acids of Venus along with introduced 5' *Nco*I and 3' *Bgl*II sites were amplified via PCR (primers Fp1905 and Rp1922) from pVenus-N1. The PCR products were digested with *Nco*I-*Bgl*II and ligated into *Nco*I-*Bgl*II-digested pSAT4-nEYFP-C1 [63] and the resulting plasmid was digested with *Bgl*II and *Sac*I and ligated with annealed complementary oligonucleotides (Fp1941 and Rp1946) coding for the myc epitope along with 5' *Bgl*II and 3' *Sac*I overhangs, yielding pSAT4/nVenus C1. pVenus-N1 (provided by Peter Kim, NIH) was constructed by disrupting (via site-directed mutagenesis) the Venus dimerization domain in pVenus-N1-NPY [62] (Nagai et al., 2002) and then cloning the resulting modified Venus gene into pEGFP-N1 (Clontech) lacking the GFP ORF. To construct pSAT4/nVenus N1, sequences encoding the N-terminal 174 amino acids of Venus, along with an introduced stop codon and 5' *Bam*HI and 3' *Xba*I sites, were amplified via PCR (primers Fp1909 and Rp1924) from pVenus-N1. PCR products were then digested with *Bam*HI and *Xba*I and ligated into *Bam*HI-*Xba*I-digested pSAT4A-nEYFP-N1 [63] that was isolated from dam^- ^*E. coli *due to dam methylation of the *Xba*I site in the plasmid's MCS. The resulting plasmid was then digested with *Sac*II and *Bam*HI and ligated with annealed complementary oligonucleotides (Fp1943 and Rp1944) coding for the myc epitope along with 5' *Sac*II and 3' *Bam*HI overhangs, yielding pSAT4/nVenus N1. pSAT4/cVenus C1 was generated by amplifying via PCR (primers Fp1907 and Rp1908 and pVenus-N1 as template DNA) sequences encoding for the C-terminal 65 amino acids (residues 175–239) of Venus, along with a start codon and 5' *Nco*I and 3' *Bgl*II sites. PCR products were digested with *Nco*I and *Bgl*II and ligated into *Nco*I-*Bgl*II-digested pSAT2-cEYFP-C1-B [63]. The resulting plasmid was then digested with *Bgl*II and *Sac*I and ligated with annealed complementary oligonucleotides (Fp1942 and Rp1947) coding for the HA eptitope tag, along with 5' *Bgl*II and 3' *Sac*I overhangs, yielding pSAT4/cVenus C1. pSAT4/cVenus N1 was generated by amplifying via PCR (primers Fp1911 and Rp1912) sequences encoding the C-terminal 65 amino acids and stop codon of Venus, along with introduced 5' *Bam*HI and 3' *Xba*I sites. PCR products were then digested with *Bam*HI and *Xba*I and ligated into dam^- ^*Bam*HI-*Xba*I-digested pSAT4A-cEYFP-N1 [63]. The resulting plasmid was digested with *Sac*II and *Bam*HI and ligated with annealed complementary oligonucleotides (Fp1945 and Rp1948) coding for the HA epitope along with 5' *Sac*II and 3' *Bam*HI overhangs, yielding pSAT4/cVenus N1.

Construction of BiFC plasmids containing TOM20 (or the mutant version thereof; TOM20mut), TOM22, TOM40 or METAXIN fused at their N termini to nVenus was carried out as follows. First, sequences encoding the entire TOM20, TOM22, TOM40 or METAXIN ORFs were amplified via PCR using the appropriate oligonucleotide primers (Additional file 4). Plasmids used as template DNA in these PCRs included pRTL2/myc-TOM20-III (provided by P. Dhanoa, University of Guelph), encoding a N-terminal myc-tagged version of Arabidopsis TOM20 isoform III, pSPORT1/TOM22-I (ABRC, clone U83367), encoding isoform I of Arabidopsis TOM22, pUNI51/TOM40-I (ABRC, clone U11102), encoding isoform I of Arabidopsis TOM40, and pUNI51/METAXIN (ABRC, clone U2140), encoding Arabidopsis METAXIN. All PCRs also introduced 5' *Eco*RI and 3' *Xma*I sites. Resulting PCR products were then digested with *Eco*RI and *Xma*I and fragments were ligated into *Eco*RI-*Xma*I-digested pSAT4/nVenus C1, yielding pSAT4/nVenus-TOM20, pSAT4/nVenus-TOM22, pSAT4/nVenus-TOM40 and pSAT4/nVenus-METAXIN. pSAT4/nVenus-TOM20mut was generated by removing, via site-directed mutagenesis (primers Fp2274 and Rp2275), sequences encoding the first 142 amino acid residues of TOM20 in pSAT4/nVenus-TOM20. This N-terminal region of TOM20 contains a tetratricopeptide motif-based receptor domain proposed to interact with mitochondrial presequences [58].

To construct BiFC plasmids containing p36 fused at its C terminus to nVenus, sequences encoding the entire p36 ORF was amplified via PCR using the appropriate oligonucleotide primers (Additional file 4) and pRTL2/p36-myc as template DNA. The PCR also introduced 5' *Eco*RI and 3' *Xma*I sites. Resulting PCR products were then digested with *Eco*RI and *Xma*I and fragments were ligated into *Eco*RI-*Xma*I-digested pSAT4/nVenus C1, yielding pSAT4/p36-nVenus.

pSAT4/mtOM64-nVenus, consisting of the Arabidopsis mitochondrial outer membrane protein of 64-kD (mtOM64), a protein proposed to function as mitochondrial protein receptor [60,52] and fused at its C terminus to nVenus, was constructed in the following manner. Initially, a cDNA encoding mtOM64 was isolated using a reverse-transcription PCR-based cloning procedure. Total RNA was purified from (50 mL) of 4-day-old Arabidopsis (*Arabidopsis thaliana *var. *Landsberg erecta*) suspension-cultured cells using Tri Reagent (Sigma-Aldrich Ltd.) and cDNA was then synthesized using an oligo(dT)_18 _primer and the RevertAid™ H Minus First Strand cDNA Synthesis Kit (Fermentas, Burlington, Canada). cDNA was then used as a template in a PCR that included primers (Fp2096 and Rp2097) designed specifically to amplify the entire mtOM64 ORF along with 5' *Xho*I and 3' *Xma*I sites. PCR conditions consisted of a 2 min incubation at 94°C, followed by 35 cycles of 40 sec at 94°C, 40 sec at 55°C, and 30 sec at 72°C, and then a 10-minute extension at 72°C. PCR products were ligated into pCR2.1 TOPO, yielding pCR2.1 TOPO/mtOM64. Next, the *Xho*I-*Xma*I fragments from pCR2.1 TOPO/mtOM64 was ligated into *Xho*I-*Xma*I-digested pSAT4/nVenus C1, yielding pSAT4/mtOM64-nVenus.

Construction of BiFC plasmids containing the βATPase presequence, porin, p36, or p33 fused at their C termini to cVenus was carried out as follows. Sequences encoding either the βATPase mitochondrial targeting presequence or the entire ORF of porin, p36 or p33 were amplified via PCR using the appropriate oligonucleotide primers (Additional file 4). Plasmids used as template DNA in these PCRs included pUC18/βATPase-GFP (see below 'Other plasmids'), pUNI51/porin (ABRC, clone U12693) encoding the Arabidopsis porin (also referred to as voltage-dependent anion-selective channel protein 1), pRTL2/p36-myc, and pRTL2/myc-p33 [25]. PCRs with plasmids encoding porin, p36 and p33 also introduced a 5' *Eco*RI site and a 3' *Xma*I site that removed each protein's stop codon, whereas PCR with βATPase presequence-containing plasmid DNA introduced 5' and 3' *Bam*HI sites. Resulting PCR products were then digested with *Eco*RI and *Xma*I or *Bam*HI and fragments were ligated into *Eco*RI-*Xma*I- or *Bam*HI-digested pSAT4/cVenus N1, yielding pSAT4/βATPase-cVenus, pSAT4/porin-cVenus, pSAT4/p36-cVenus and pSAT4/p33-cVenus. To construct pSAT4/cVenus-Cb5 consisting of the mitochondrial isoform (isoform D) of tung Cb5 [27] fused at its N terminus to cVenus, the entire ORF of Cb5 along with 5' *Eco*RI and 3' *Xba*I sites was amplified by PCR (primers Fp497 and Rp380) from pRTL2/myc-Δ*Nhe*I Cb5D [27]. PCR products were then digested with *Eco*RI and *Xba*I and ligated into *Eco*RI/*Xba*I-digested pSAT4/cVenus C1, yielding pSAT4/cVenus-Cb5.

#### Other Plasmids

pUC18/βATPase-GFP encoding the N-terminal mitochondrial matrix targeting presequence (residues 1–60) of the β subunit of F_1_-ATPase from *Nicotiana plumaginfolia *fused to the N terminus of GFP was constructed by amplifying via PCR (primers FpRD1 and RpRD2) the βATPase presequence along with 5' and 3' *Nhe*I sites, and using pBIN35Sβ60catE9 (provided by F. Chaumont, University of Louvain) [66] as the template DNA. The resulting PCR products were then digested with *Nhe*I and ligated into *Nhe*I-digested pUC18/*Nhe*I-GFP, a modified version of pUC18/GFP [88] containing an in-frame *Nhe*I site immediately upstream of the GFP ORF. The construction of pRTL2/RFP, also referred to as pRTL2/MCS-RFP-stop, has been described elsewhere [89]. pRTL2/RFP-MFP encoding the RFP fused to the entire ORF of peroxisomal multifunctional protein (MFP) was constructed by ligating the *Xba*I fragment from pRTL2/GFP-MFP [90] into *Xba*I-digested pRTL2-RFP-MCS [89].

### Transient transformation of tobacco BY-2 cells and fluorescence microscopy

Tobacco (*Nicotiana tabacum *cv BY-2) suspension-cultured cells were maintained and prepared for biolistic bombardment as described previously [19]. Briefly, all transient transformations, with the exception of those for BiFC (see below), were performed using tungsten particles coated with 10 μg of plasmid DNA (or 5 μg of each plasmid for co-transformations) and a biolistic particle delivery system-1000/HE (Bio-Rad Laboratories, Mississauga, Canada). Bombarded cells were incubated for 4 h to allow for expression and sorting of the introduced gene product(s), then fixed in formaldehyde, incubated with 0.01% (w/v) pectolyase Y-23 (Kyowa Chemical Products, Osaka, Japan), and permeabilized with either 0.3% (v/v) triton X-100 or 25 μg mL^-1 ^digitonin (Sigma-Aldrich Ltd.) [26]. Cells were evaluated after 4 h to ensure that any potential negative effects due to (membrane) protein over-expression were avoided.

Fixed and permeabilized cells were processed for immunofluorescence microscopy as described by Trelease et al. [91]. Primary and dye-conjugated secondary antibodies and sources were as follows: mouse anti-myc antibodies in hybridoma medium (clone 9E10; Princeton University, Monoclonal Antibody Facility, Princeton, NJ, USA); mouse anti-CAT antibodies in hybridoma medium (provided by S. Subramani, University of California, San Diego); rabbit anti-cottonseed catalase IgGs [92]; mouse anti-maize porin [24]; rabbit anti-pea E1β [93]; mouse anti-α-tubulin (clone DM 1A) (Sigma-Aldrich Ltd.); rabbit anti-myc and mouse anti-HA (Bethyl Laboratories, Montgomery, TX, USA); rabbit anti-p36 antibodies were raised against a synthetic peptide corresponding to an identical amino acid sequence in both p36 and TBSV p33 (-VEPARELKGKDGEDLLTGSR-) (residues 218–237 in p36 and 184–203 in p33) (refer to the p33 and p36 sequence alignment shown in Figure 4A) [25]; goat anti-mouse Alexa Fluor 488 IgGs (Cedar Lane Laboratories, Ontario, Canada); goat anti-rabbit rhodamine red-X IgGs (Jackson ImmunoResearch Laboratories, West Grove, PA, USA). Concanavalin A conjugated to Alexa 594 (Molecular probes, Eugene, OR) was added to BY-2 cells at a final concentration of 5 μg mL^-1 ^during the final 20 min of incubation with secondary antibodies. All antibodies raised in rabbits were IgG-affinity purified using protein A-Sepharose columns.

Epifluorescent images of BY-2 cells were acquired using a Zeiss Axioskop 2 MOT epifluorescence microscope (Carl Zeiss, Toronto, Canada) with a Zeiss 63× Plan Apochromat oil-immersion objective. Image captures were performed using a Retiga 1300 charge-coupled device camera (Qimaging, Burnaby, Canada) and Northern Eclipse 5.0 (Empix Imaging, Mississauga, Canada) or OpenLab 5.0 (Improvision Inc., Lexington, MA, USA) software. CLSM images of BY-2 cells were acquired using a Leica DM RBE microscope with a Leica 63× Plan Apochromat oil-immersion objective, a Leica TCS SP2 scanning head, and the Leica TCS NT software package (Version 2.61) (Leica, Heidelberg, Germany). Fluorophore emissions were collected sequentially in double- and triple-labeling experiments; single-labeling experiments showed no detectable crossover at the settings used for data collection. Confocal images were acquired as a z-series of representative cells and single optical sections were saved as 512 × 512-pixel digital images. All fluorescence images of cells shown in individual figures are representative of > 50 independent (transient) transformations from at least two independent transformation experiments. Figure compositions were generated using Adobe Photoshop CS (Adobe Systems, San Jose, CA).

### BiFC

Biolistic bombardment of cells for BiFC experiments was carried out essentially as described above with the exception that tungsten particles were coated with 200 ng each of plasmids encoding the appropriate combinations of proteins. Tungsten particles were coated also with 200 ng of pRTL2/RFP serving as an internal reference marker to help identify cells that were transformed and, thus, could be examined for the BiFC signal. Alternatively, particles were coated also with 200 ng of pUC18/βATPase-GFP or pRTL2/RFP-MFP serving as a mitochondrial or peroxisomal marker protein, respectively, and for assessing (mitochondrial or peroxisomal) localization of co-expressed Venus half fusion proteins.

Approximately 16 h after bombardment, cells were formaldehyde fixed and either examined (via fluorescence microscopy) for BiFC (and RFP) or processed for immunofluorescence microscopy as described above. The 16 h time point and the amounts of plasmid DNA employed in BiFC assays were chosen based on preliminary optimization experiments aimed at eliminating the possibility of any non-specific (false positive) reconstitutions that may occur due to protein (over)expression [reviewed in [94,95]]. Overall, the conditions used for BiFC were based on those in which nVenus- or nVenus-tagged versions of the various TOM proteins, p36, p33, porin and the βATPase presequence co-expressed with the corresponding "empty" vector containing the nVenus or cVenus alone yield no significant BiFC (Venus) fluorescence (data not shown). Also, transformations with RFP (or βATPase-GFP or RFP-MFP) alone, confirmed that there was no cross-bleed of the red (or green) fluorescence signal into the yellow channel.

Topological orientations of Venus half fusion proteins were determined using digitonin (differential) permeabilization as described above, with the exception that biolistic bombardments were performed with gold particles (Sigma-Aldrich Ltd.) coated with 5 μg of plasmid DNA, and cells were formaldehyde fixed ~4 h after bombardment.

### Rub inoculation of C. quinoa and electron microscopy

*Chenopodium quinoa *plants were grown in chambers at 21°C with a 12 h light-12 h dark cycle and five days prior to rub inoculation plants were transferred to a laboratory bench top with lower light conditions to facilitate the infection process [86]. Rub inoculations (including mock inoculations) were carried out with 6-to-8-leaf stage plants and 10 μg of CIRV infectious cDNA diluted in 30 μl of inoculation buffer [86]. Approximately 7 to 10 days after inoculations, ~1 cm^2 ^sections of the rub-innoculated leaf, including several necrotic lesions, were removed and processed for electron microscopy.

CIRV cDNA rub-inoculated *C. quinoa *leaf pieces were processed for transmission electron microscopy (TEM) by fixing in 4% (v/v) gluteraldehyde (Fisher Scientific, Markam, Canada) and 1% (v/v) acrolein (Sigma-Aldrich Ltd.) in 0.025 M NaKPO_4 _buffer (pH 7.2) for 2 h in a vacuum chamber and then at 4°C overnight. Samples were then washed three times in 0.025 M NaKPO_4 _buffer at 2 h intervals and 4°C, then post-fixed overnight at 4°C in 1% (w/v) osmium tetroxide (Fisher Scientific), dehydrated in a graded series of ethanol (50 to 100% [v/v]), and embedded in Spurr's medium-grade epoxy [96]. Thin sections were cut with a glass or diamond knife, mounted on copper grids and post-stained with 2% (w/v) uranyl acetate and Reynold's lead citrate [97] for 10 and 3 min, respectively. All images were acquired at 80 kV with a Phillips (Mahwah, NJ, USA) CM10 TEM.

### Accession numbers

National Center for Biotechnology Information (NCBI) and/or Arabidopsis Genome Initiative (AGI) database accession numbers for proteins described in this study are as follows: βATPase (P17614), Cb5D (AAT84461), CIRV p36 (CAA59477), CIRV p95 (NP_612580), METAXIN (NP_565446, At2g19080), MFP (AAL35606), mtOM64 (NP_196504; At5g09420), porin (Q9SRH5; At3g01280), TBSV p33 (NP_062898), TOM20 (NP_189344; At3g27080), TOM22 (NP_563699; At1g04070), TOM40 (NP_188634; At3g2000).

## Abbreviations

βATPase: β subunit of the F_1_-ATPase; BiFC: bimolecular fluorescence complementation; BY-2: Bright Yellow-2; CAT: chloramphenicol acetyltransferase; Cb5: cytochrome *b*_5_; CIRV: *Carnation Italian ringspot virus*; ConA: Concanavalin A; CLSM: confocal laser-scanning microscopy; CaMV: Cauliflower Mosaic Virus; cVenus: C-terminal half of Venus; CymRSV: *Cymbidium ringspot virus*; E1β: β subunit of the mitochondrial pyruvate dehydrogenase complex; ER: endoplasmic reticulum; FIS1: protein, fission 1; Fzo1: fuzzy onion 1; GFP: green fluorescent protein; HA: hemaglutinin; kD: kilodalton; MCS: multiple cloning site; Mfn: mitofusin; MFP: multifunctional protein; mtOM64: mitochondrial outer membrane protein of 64-kD; MVB: multivesicular body; ORF: open reading frame; pER: peroxisomal ER; PCR: polymerase chain reaction; FIS1: protein fission 1; nVenus: N-terminal half of Venus; p33: 33-kD membrane-bound replication protein; p36: 36-kD RNA-binding protein; p92: 92-kD RNA-dependent RNA polymerase; p95: 95-kD RNA-dependent RNA polymerase; RFP: red fluorescent protein; SAM: sorting and assembly machinery; TBSV: *Tomato bushy stunt virus*; TEM: transmission electron microscopy; TIM: translocase of the inner mitochondrial membrane; TOM: translocase complex in the mitochondrial outer membrane; TMD: transmembrane domain; YFP: yellow fluorescent protein.

## Authors' contributions

YTH, AWM and SKG conducted the experiments. YTH, AWM and RTM designed and planned the experiments, YTH and RTM were involved in writing the paper, and all authors read, provided critical comments, and approved the final manuscript.

## Supplementary Material

Additional file 1 **Localization of p36 expressed alone or together with the p95 replication protein in BY-2 cells and electron microscopic analysis of *Chenopodium quinoa *meosphyll cells transformed with CIRV**. **(A) **Tobacco BY-2 cells were transformed transiently (via biolistic bombardment) with p36 either alone, together with the other CIRV replication protein p95 (Rep), or in the context of full-length infectious CIRV (CIRV cDNA). Cells were then processed for immunofluorescence CLSM with expressed p36 and p95 being immunodetected using primary antibodies raised against a synthetic peptide that corresponds to an amino acid sequence in both p36 and p95 [25]. As mentioned in the 'Introduction', p95 is produced by the translational read-through of the p36 amber stop codon [7]. Hatched boxes represent the portion of the cells shown at higher magnification in the panels to the right. The yellow/orange color in the merge images indicate co-localization of expressed p36 (and p95) and the endogenous outer mitochondrial membrane protein porin; arrowheads also indicate obvious co-localizations. Bar = 10 μm. **(B) **Individual *C. quinoa *leaves rub-inoculated with an infectious CIRV cDNA (see 'Methods and Materials' for details) or mock rub-inoculated at 7 days post inoculation. Arrowheads indicate examples of necrotic lesions on the surface of the CIRV-infected leaf; necrotic lesions were not observed on leaves of mock rub-inoculated plants. Bar = 2 cm. **(C) **Representative transmission electron micrographs of a mitochondria-derived MVB and wild-type mitochondrion in mesophyll cells of *C. quinoa *leaves rub-inoculated with the infectious cDNA of CIRV and mock rub-inoculated, respectively. Arrowheads denote examples of distinct vesicle/spherule-like structures located in the intermembrane space of the mitochondria-derived MVB that are proposed to be derived by invaginations of the outer mitochondrial membrane and serve as the sites for CIRV RNA replication [13,4]. Note also that the cristae are significantly altered (and less in number) in the mitochondria-derived MVB of the CIRV-transformed cell; compare with morphology of the cristae in the mitochondria of the mock-transformed cell. CW, cell wall; Cyt, cytosol; Mito, mitochondria; mMVB, mitochondria-derived multivesicular body; Vac, vacuole. Bars = 0.5 μm. Click here for file

Additional file 2 **Localization of p36-CAT and topological orientation of p36 90-190-CAT in BY-2 cells**. **(A) **Tobacco BY-2 cells were transformed transiently (via biolistic bombardment) with p36 90-190-CAT (consisting of the p36 amino acid residues 90–190 fused to the N-terminus of CAT) and then processed for immunofluorescence CLSM using primary antibodies raised against CAT. Hatched boxes represent the portion of the cells shown at higher magnification in the panels to the right. The merged image shows that the torus fluorescent structures containing p36 90-190-CAT delineate the spherical structures attributable to mitochondrial matrix-localized E1β. Arrowheads indicate obvious examples of a toroidal enclosure of a sphere. Bar = 10 μm. **(B) **BY-2 cells were transformed transiently (via biolistic bombardment) with p36 90-190-CAT, fixed, and then permeabilized with either triton X-100 (which permeabilizes both the plasma membrane and organellar membranes) or digitonin (which permeabilizes only the plasma membrane). Permeabilized cells were then processed for (immuno)epifluorescence microscopy using antibodies raised against (as indicated by the labelling at the top left of each micrograph) either cytosolic α-tubulin, mitochondrial matrix E1β, or CAT (in p36 90-190-CAT). Note that, similar to endogenous cytosolic α-tubulin, p36 90-190-CAT, but not endogenous mitochondrial matrix E1β, were immunodetected in both triton X-100- and digititon-permeabilized cells, indicating that the C-terminal-appended CAT moiety was exposed to the cytosol. Although the relative position of the N terminus of p36 90-190-CAT was not directly tested in these differential permeabilization experiments, this fusion protein, similar to full-length p36 (refer to Figure 2), is likely orientated in an N_out_-C_out _topology. This is because the cytosolic-facing C terminus of p36 90-190-CAT, together with an even number (two) of predicted TMDs, suggests that its N terminus is also exposed to the cytosol. Differential interference contrast (DIC) images correspond to the same cells shown to the left. Bar = 10 μm. Click here for file

Additional file 3 **Localization and topology of nVenus and cVenus fusion proteins used in BiFC assays**. **(A) **Tobacco BY-2 cells were transformed transiently (via biolistic bombardment) with selected individual nVenus (and myc-tagged) or cVenus (and HA-tagged) fusion proteins as shown in Figures 7A and 7B (refer to Methods *'Construction of plasmids: Plasmids used for BiFC' *for details on the cloning of individual Venus half and epitope-tagged fusion proteins). With the exception of cell transformed with p33-cVenus, all cells were also co-transformed with βATPase-GFP, consisting of the N-terminal mitochondrial targeting presequence (residues 1–60) of the βATPase fused to the N terminus of GFP, and serving as a well-established mitochondrial marker protein [66,67], and thus confirming their mitochondrial localization. Cells transformed with p33-cVenus were co-transformed with RFP-MFP, consisting of the RFP fused to the N-terminal end of peroxisomal matrix marker protein MFP serving as a peroxisomal matrix marker protein [90,99]. At ~16 h post-bombardment, all cells were then processed for (immuno)epifluorescence microscopy using anti-myc or anti-HA antibodies. Each micrograph is labelled at the top left with the name of the transiently co-expressed fusion protein. Differential interference contrast (DIC) images correspond to the same cells shown to the left. Note that with the exception of peroxisomal-localized p33-cVenus, all individual nVenus and cVenus fusion proteins sorted to mitochondria as evidenced by their co-localization with βATPase-GFP. p33-cVenus, on the other hand, colocalized with RFP-MFP, as expected for this peroxisomal-localized viral protein [25]. Bar = 10 μm. **(B) **BY-2 cells were transformed with various individual nVenus (and myc-tagged) or cVenus (and HA-tagged) fusion proteins as in (A); however, cells were incubated with digitonin (rather than triton X-100 as in [A]) which permeabilizes only the plasma membrane and not organellar membranes [26]. Permeabilized cells were then processed for immuno(epi)fluorescence microscopy using anti-myc, anti-HA, anti-E1β, anti-βATPase and/or anti-α-tubulin antibodies. Each micrograph is labelled at the top left with the name of the transiently-expressed fusion protein or endogenous E1β, βATPase or α-tubulin. Note that the presence or absence of an immunofluorescence signal attributable to an expressed fusion protein indicates whether or not the protein's appended myc- or HA-epitope tag (immediately adjacent to nVenus or cVenus, respectively) is exposed to the cytosol or not; compare to the absence or presence of an immunofluorescence signal attributable to mitochondrial matrix-localized E1β or cytosolic α-tubulin in the same cells. Note also that the relative position of the myc- or HA-epitope tag (and the immediately adjacent nVenus or cVenus fragment, respectively) for selected fusion proteins localized to the mitochondrial outer membrane are shown in Figure 7B (indicated with asterisks). DIC images correspond to the same cells shown to the left. Bar = 10 μm. Click here for file

Additional file 4 List of synthetic oligonucleotide primers used in the construction of plasmids. Click here for file
