# Supplementary figures and images for: Localization of the Carnation Italian ringspot virus replication protein p36 to the mitochondrial outer membrane is mediated by an internal targeting signal and the TOM complex
Source: BMC Cell Biol. 2008 Sep 23;9:54. doi: 10.1186/1471-2121-9-54 (PMC2573885; doi:10.1186/1471-2121-9-54)

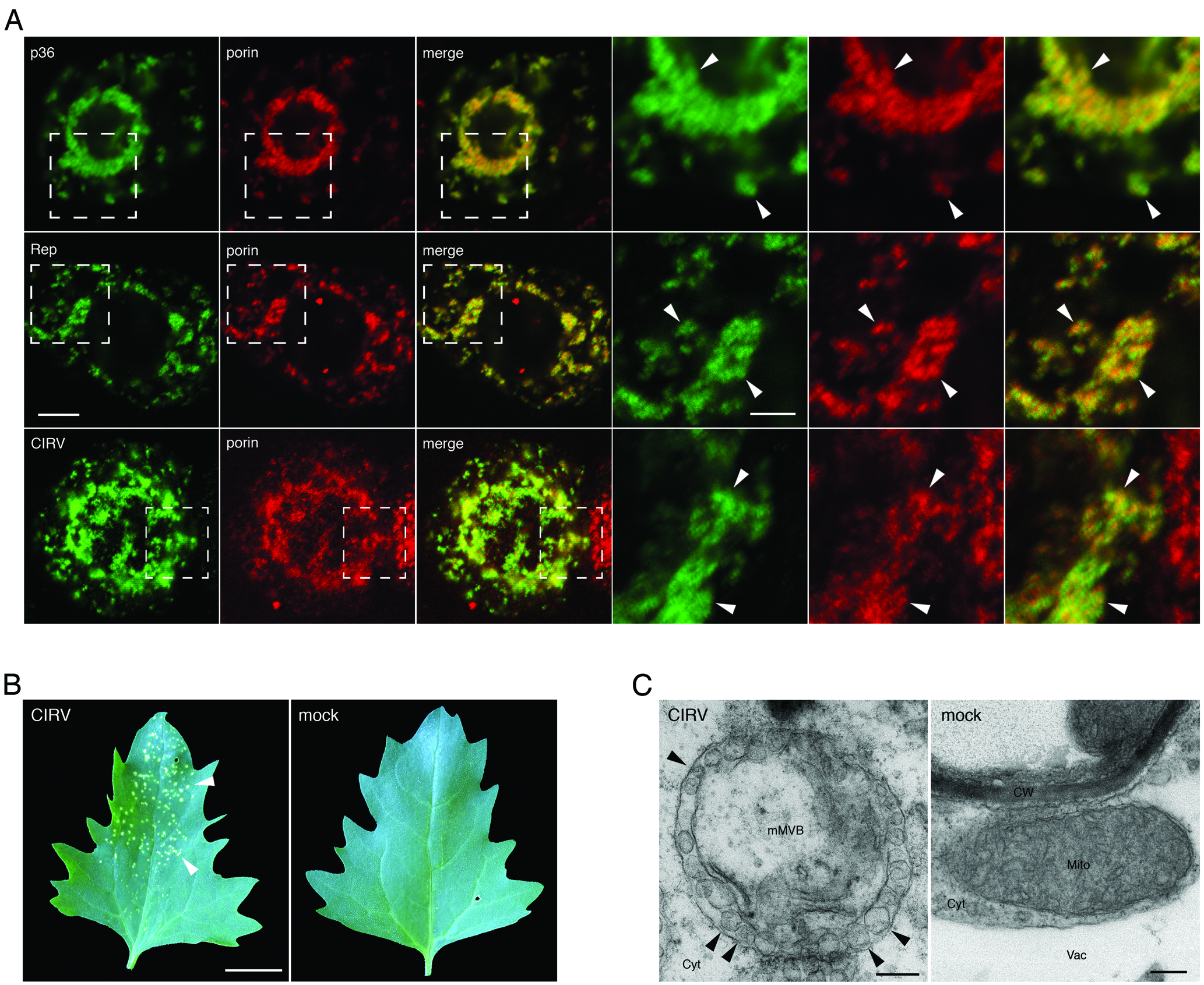

Supplement: Additional file 1 — Localization of p36 expressed alone or together with the p95 replication protein in BY-2 cells and electron microscopic analysis of Chenopodium quinoa meosphyll cells transformed with CIRV. (A) Tobacco BY-2 cells were transformed transiently (via biolistic bombardment) with p36 either alone, together with the other CIRV replication protein p95 (Rep), or in the context of full-length infectious CIRV (CIRV cDNA). Cells were then processed for immunofluorescence CLSM with expressed p36 and p95 being immunodetected using primary antibodies raised against a synthetic peptide that corresponds to an amino acid sequence in both p36 and p95 [25]. As mentioned in the 'Introduction', p95 is produced by the translational read-through of the p36 amber stop codon [7]. Hatched boxes represent the portion of the cells shown at higher magnification in the panels to the right. The yellow/orange color in the merge images indicate co-localization of expressed p36 (and p95) and the endogenous outer mitochondrial membrane protein porin; arrowheads also indicate obvious co-localizations. Bar = 10 μm. (B) Individual C. quinoa leaves rub-inoculated with an infectious CIRV cDNA (see 'Methods and Materials' for details) or mock rub-inoculated at 7 days post inoculation. Arrowheads indicate examples of necrotic lesions on the surface of the CIRV-infected leaf; necrotic lesions were not observed on leaves of mock rub-inoculated plants. Bar = 2 cm. (C) Representative transmission electron micrographs of a mitochondria-derived MVB and wild-type mitochondrion in mesophyll cells of C. quinoa leaves rub-inoculated with the infectious cDNA of CIRV and mock rub-inoculated, respectively. Arrowheads denote examples of distinct vesicle/spherule-like structures located in the intermembrane space of the mitochondria-derived MVB that are proposed to be derived by invaginations of the outer mitochondrial membrane and serve as the sites for CIRV RNA replication [13,4]. Note also that the cristae are significa [file 1471-2121-9-54-S1.tiff]

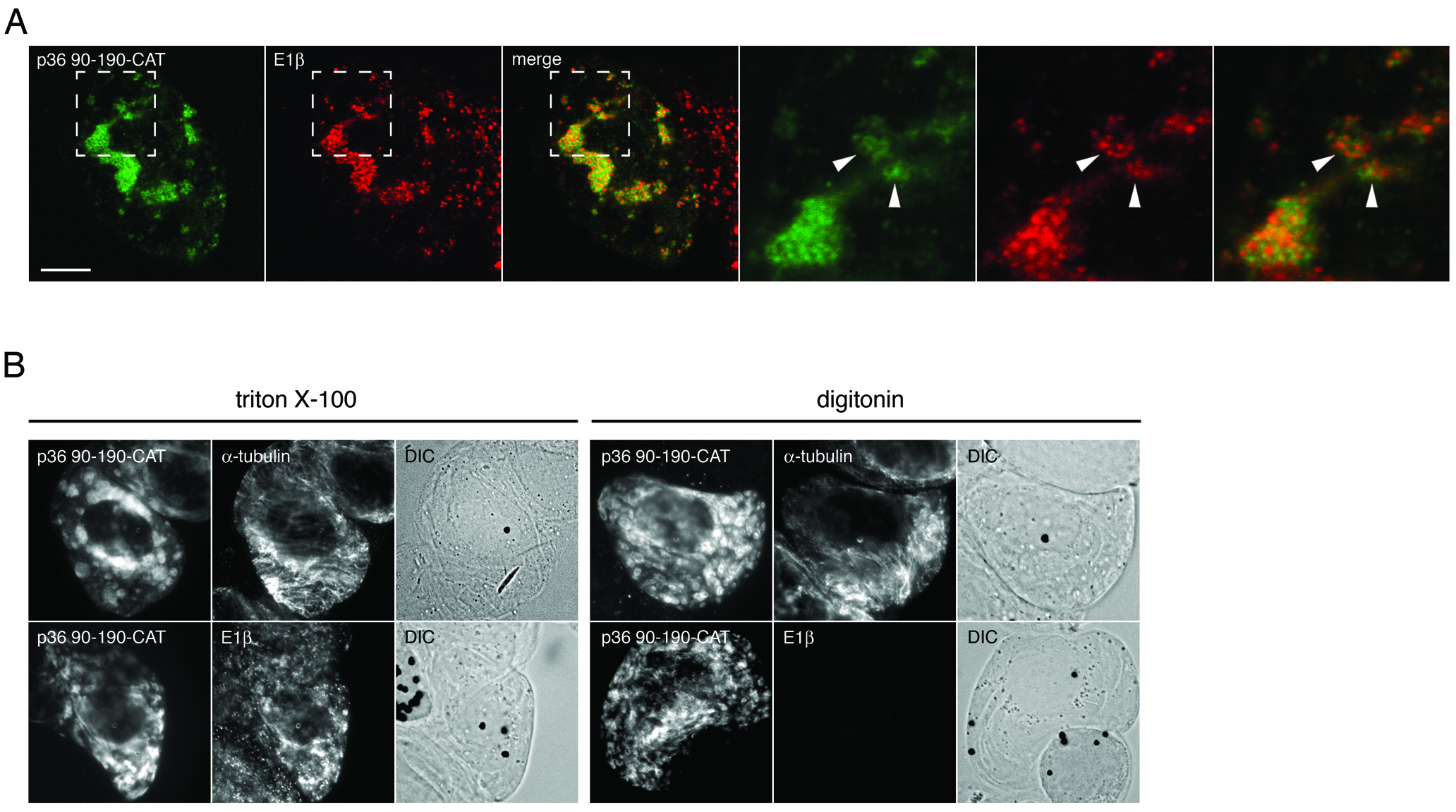

Supplement: Additional file 2 — Localization of p36-CAT and topological orientation of p36 90-190-CAT in BY-2 cells. (A) Tobacco BY-2 cells were transformed transiently (via biolistic bombardment) with p36 90-190-CAT (consisting of the p36 amino acid residues 90–190 fused to the N-terminus of CAT) and then processed for immunofluorescence CLSM using primary antibodies raised against CAT. Hatched boxes represent the portion of the cells shown at higher magnification in the panels to the right. The merged image shows that the torus fluorescent structures containing p36 90-190-CAT delineate the spherical structures attributable to mitochondrial matrix-localized E1β. Arrowheads indicate obvious examples of a toroidal enclosure of a sphere. Bar = 10 μm. (B) BY-2 cells were transformed transiently (via biolistic bombardment) with p36 90-190-CAT, fixed, and then permeabilized with either triton X-100 (which permeabilizes both the plasma membrane and organellar membranes) or digitonin (which permeabilizes only the plasma membrane). Permeabilized cells were then processed for (immuno)epifluorescence microscopy using antibodies raised against (as indicated by the labelling at the top left of each micrograph) either cytosolic α-tubulin, mitochondrial matrix E1β, or CAT (in p36 90-190-CAT). Note that, similar to endogenous cytosolic α-tubulin, p36 90-190-CAT, but not endogenous mitochondrial matrix E1β, were immunodetected in both triton X-100- and digititon-permeabilized cells, indicating that the C-terminal-appended CAT moiety was exposed to the cytosol. Although the relative position of the N terminus of p36 90-190-CAT was not directly tested in these differential permeabilization experiments, this fusion protein, similar to full-length p36 (refer to Figure 2), is likely orientated in an Nout-Cout topology. This is because the cytosolic-facing C terminus of p36 90-190-CAT, together with an even number (two) of predicted TMDs, suggests that its N terminus is also exposed to the cytosol. Differential interfe [file 1471-2121-9-54-S2.tiff]

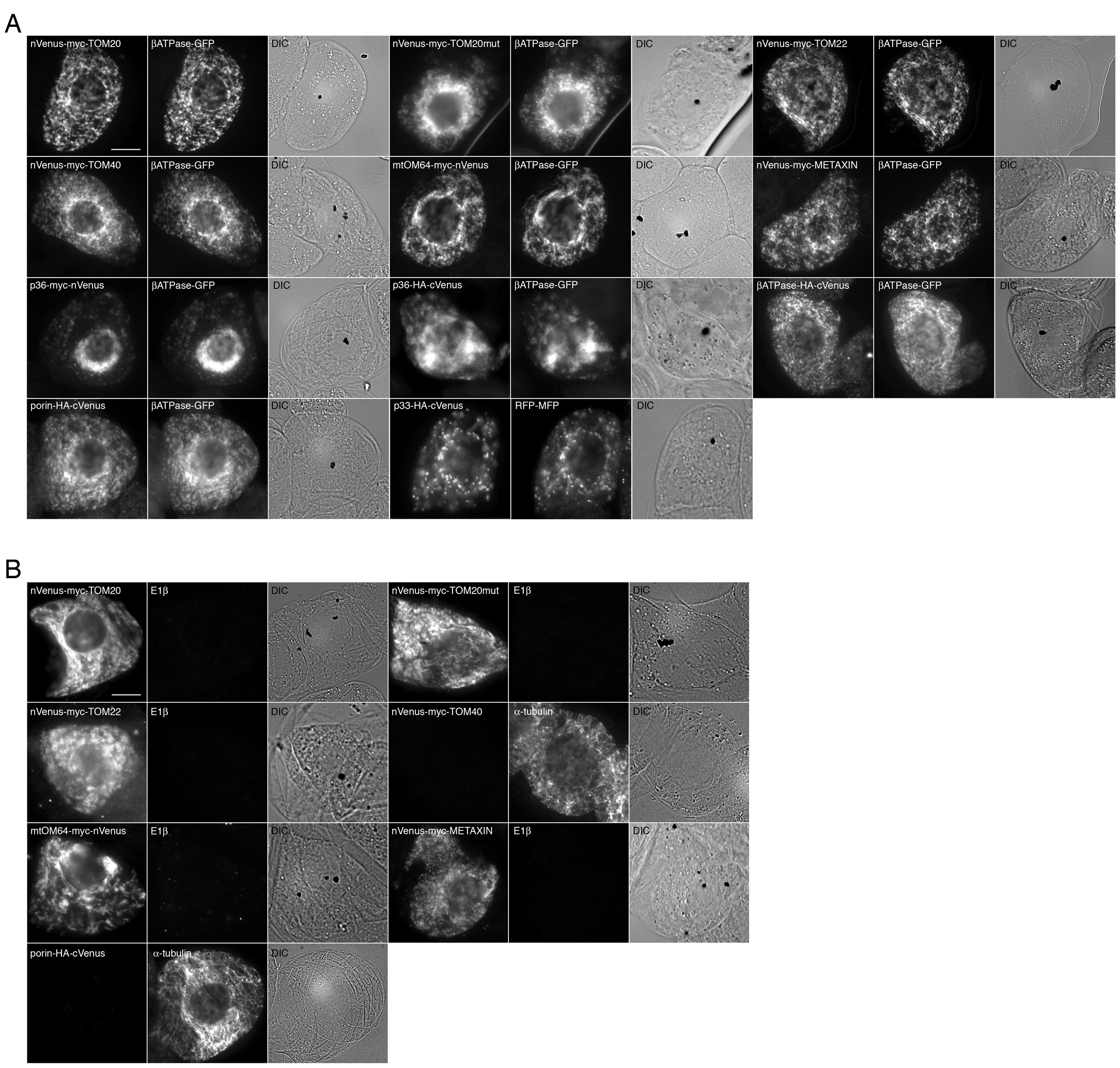

Supplement: Additional file 3 — Localization and topology of nVenus and cVenus fusion proteins used in BiFC assays. (A) Tobacco BY-2 cells were transformed transiently (via biolistic bombardment) with selected individual nVenus (and myc-tagged) or cVenus (and HA-tagged) fusion proteins as shown in Figures 7A and 7B (refer to Methods 'Construction of plasmids: Plasmids used for BiFC' for details on the cloning of individual Venus half and epitope-tagged fusion proteins). With the exception of cell transformed with p33-cVenus, all cells were also co-transformed with βATPase-GFP, consisting of the N-terminal mitochondrial targeting presequence (residues 1–60) of the βATPase fused to the N terminus of GFP, and serving as a well-established mitochondrial marker protein [66,67], and thus confirming their mitochondrial localization. Cells transformed with p33-cVenus were co-transformed with RFP-MFP, consisting of the RFP fused to the N-terminal end of peroxisomal matrix marker protein MFP serving as a peroxisomal matrix marker protein [90,99]. At ~16 h post-bombardment, all cells were then processed for (immuno)epifluorescence microscopy using anti-myc or anti-HA antibodies. Each micrograph is labelled at the top left with the name of the transiently co-expressed fusion protein. Differential interference contrast (DIC) images correspond to the same cells shown to the left. Note that with the exception of peroxisomal-localized p33-cVenus, all individual nVenus and cVenus fusion proteins sorted to mitochondria as evidenced by their co-localization with βATPase-GFP. p33-cVenus, on the other hand, colocalized with RFP-MFP, as expected for this peroxisomal-localized viral protein [25]. Bar = 10 μm. (B) BY-2 cells were transformed with various individual nVenus (and myc-tagged) or cVenus (and HA-tagged) fusion proteins as in (A); however, cells were incubated with digitonin (rather than triton X-100 as in [A]) which permeabilizes only the plasma membrane and not organellar membranes [26]. Permeabilized cells w [file 1471-2121-9-54-S3.tiff]
