# Supplementary material for: Localization of the Carnation Italian ringspot virus replication protein p36 to the mitochondrial outer membrane is mediated by an internal targeting signal and the TOM complex
Source: BMC Cell Biol. 2008 Sep 23;9:54. doi: 10.1186/1471-2121-9-54 (PMC2573885; doi:10.1186/1471-2121-9-54)
Supplement: Additional file 4 — List of synthetic oligonucleotide primers used in the construction of plasmids. [file 1471-2121-9-54-S4.pdf]

Additional file 4 – List of synthetic oligonucleotide primers used in the construction of plasmids.

Plasmid	Primers	Sequence (5' to 3')
pRTL2/p36	Fp135	GGCGGGCCATGGAGGGTTTGAAGGCTGAGTCTACCACAACTGTGGGG
	Rp107	GGTAGACGCACTAGGCCCCCGGGTTTGACACCGAGGGATTCC
	Fp447	GGAATCCCTCGGTGTCAAATAGCCCGGGGATCCTCTAGAGTCC
	Rp448	GGACTCTAGAGGATCCCCGGGCTATTTGACACCGAGGGATTCC
pRTL2/p36-myc	Fp177	CCGGCGAACAAAAGTTGATTTCTGAAGAAGATTTGTAGAGATCTC
	Rp178	CCGGGAGAATCTCTACAAATCTTCTTCAGAAATCAACTTTTGTTCG
pRTL2/myc-p36	Fp29	CATGGAACAAAAGTTGATTTCTGAAGAAGATTTGGG
	Rp30	CATGCCGGCGTAGTCTGGCACGTCGTATGGGTACCC
pRTL/Rep	Fp135	GGCGGGCCATGGAGGGTTTGAAGGCTGAGTCTACCACAACTGTGGGG
	Rp572	CCCGCGCCCGGGTCAAGCTACGGCGGAGTCGAGGATGCTGGGC
pRTL2/p95	Fp642	GGAATCCCTCGGTGTCAAATATGGAGGCCTAGTGCGTCTACC
	Rp641	GGTAGACGCACTAGGCCTCCATATTTGACACCGAGGGATTCC
pHST/CIRV	Fp646	GGAAGTTCATTTCATTTGGAGAGGCCTATTCTCCAGGATTCCTCGACC
	Rp645	GGTCGAGGAATCCTGGAGAATAGGCCTCTCCAAATGAAATGAACTTCC
	Fp735	GGCGGCAGGCCTAGGAAATATCTCAGGATTTGACCGTCCGGTGAGTTGCGC
	Rp734	GCCCCGCAGCACAAATAGGAACAAGTGCTGCAGCCG
pRTL2/p36-myc (*Mlu*I)	Fp275	CCCGGCGATTGTGGCAAACACGCGTATTGTTACTGGTACATGTGAGG
	Rp276	CCTCACATGTACCAGTAACAATACGCGTGTTTGCCACAATCGCCGGG
pRTL2/p36-myc (*Mlu*I/*Avr*II)	Fp277	GGCATTGCGTACGCTACCCTCCCTAGGGAAAAACTGTCTGTGTTTAGG
	Rp278	GGCATTGCGTACGCTACCCTCCCTAGGGAAAAACTGTCTGTGTTTAGG
pRTL2/p36-*Avr*II	Fp340	CCTCGGTGTCAAACCTAGGGGCCTAGTGC
	Rp341	GCACTAGGCCCCTAGGTTTGACACCGAGG
pRTL2/p36 120-190-CAT	Fp331	CCCGGCGATTGTGGCAAACCCATGGATTGTTACTGGTACATGTGAGG
	Rp332	CCTCACATGTACCAGTAACAATCCATGGGTTTGCCACAATCGCCGGG
pRTL2/p36 120-164-CAT	Fp407	CCACGTGTAGACATGAGTGCGCGTGAGAAAAAAATCACTGGATATACC
	Rp408	GGTATATCCAGTGATTTTTTTCTCACGCGCACTCATGTCTACACGTGG
pRTL2/p36 90-164-CAT	Fp449	CGGGCCATGGATTGGTTGGCTAAACGGGGTGTGGCTCGTGATTACCGC
	Rp452	CCCCCCCCTAGGACGCGCACTCATGTCTACACGTGGATAAAAGTACGG
pRTL2/p36 90-190-CAT	Fp457	CCCGCCCCTAGGCCTGGGGAGGGTAGCGTACGCAATGCC
	Rp453-2	GGGCCCGGATCCACACGCATTGTTACTGGTACATGTGAGGTGGCAGGGG
pUC18/p36 90-190-mGFP	Fp1623	GCGGGCCCATGGATTGGTTGGCTAAACGGGGTGTGGC
	Rp1624	GGGGGGCCATGGGCCTGGGGAGGGTAGCGTACGCAATGCC
	Fp1255	CGATCACATGGTCCTTAAGGAGTTCGTGACCGCC
	Rp1256	GGCGGTCACGAACTCCTTAAGGACCATGTGATCG
pRTL2/p33-myc (*Mlu*I)	Fp273	CCGCTGTTGAGTTATGCCACGCGTGTACGCGCAGTCTCAGTCAAGG
	Rp274	CCTTGACTGAGACTGCGCGTACACGCGTGGCATAACTCAACAGCGG
pRTL2/p33 103-131 p36-myc	Fp1983	CGCGTGTACGCGCAGTCTCAGTCAAGGCTTTTGGCAATGAACTATCGTT<br>CAATGTCAGGGTGCCTAGACCATCTGTACCTAAGAAAG
	Rp1984	TCGACTTTCTTAGGTACAGATGGTCTAGGCACCCTGACATTGAACGATA<br>GTTCATTGCCAAAAGCCTTGACTGAGACTGCGCGTACA
	Fp2007	GACCATCTGTACCTAAGAAAGGCATGCTCATTGGCCTGGCG
	Rp2008	CGCCAGGCCAATGAGCATGCCTTTCTTAGGTACAGATGGTC
pRTL2/p36-myc (*Mlu*I/*Sal*I/*Avr*II)	Fp576	CGTACTTTTATCCACGTGTCGACATGAGTGCGCGTGGCATGC
	Rp575	GCATGCCACGCGCACTCATGTCGACACGTGGATAAAAGTACG
pRTL2/p36-myc TMD1ΔTMD2	Fp654	GGCATGCTCATTGGCCTGGCGGCGGCTGCAGCACTTGTTCCTATTTGT<br>GCTGGCATTGCGTACGCTA
	Rp653	CGCGTAGCGTACGCAATGCCAGCACAAATAGGAACAAGTGCTGCAGCC<br>GCCGCCAGGCCAATGAGCATGCC
pRTL2/p36-myc TMD1ΔTMD2-*Sal*I	Fp576	CGTACTTTTATCCACGTGTCGACATGAGTGCGCGTGGCATGC

	Rp575	GCATGCCACGCGCACTCATGTCGACACGTGGATAAAAGTACG
pRTL2/p36-myc TMD1⇔TMD2	Fp682	TCGACATGAGTGCGCGTTACCGCACTGCCGTGGGGTTGG
	Rp680	ATGGCCAACCCCACGGCAGTGCGGTAACGCGCACTCATG
	Fp681	CCATCATACCTGGTATCCCGGCGATTGTGGCAAACACACTCC
	Rp679	CTAGGGAGTGTGTTTGCCACAATCGCCGGGATACCAGGTATG
pRTL2/p36-myc TMD1ΔsynTMD	Fp1670	CGCCTGGCCTTAGTGCTCGCACTAGTTTTGGCGCTGGTCTTAGCTCTCGTACTAA
	Rp1672	CGCGTTAGTACGAGAGCTAAGACCAGCGCCAAAACTAGTGCGAGCACTAAGGCCAGGCG
pRTL2/p36-myc TMD2ΔsynTMD	Fp1674	TCGACATGAGTGCGCGTGGCATGCTGGCCTTAGTGCTCGCACTAGTTTTG<br>GCGCTGGTCTTAGCTCTCGTACTAGCCTTGGTGCTGGCAC
	Rp1675	CTAGGTGCCAGCACCAAGGCTAGTACGAGAGCTAAGACCAGCGCCAAAAC<br>TAGTGCGAGCACTAAGGCCAGCATGCCACGCGCACTCATG
pRTL2/p36-myc TMD1ΔCb5TMD	Fp1977	CGTGCAGTTCCTGTAGCCATTGTTGGTATATCTGTGGTTGGCTTCTTATACC<br>TAA
	Rp1978	CGCGTTAGGTATAAGAAGCCAACAACCACAGATATACCAACAATGGCTACAG<br>GAACTGCACG
pRTL2/p36-myc TMD2ΔCb5TMD	Fp1979	TCGACATGAGTGCGCGTGGAGCAGTTCCTGTAGCCATTGTTGGTATATCTG<br>TGGTTGTTGGCTTCTTATACCTAC
	Rp1980	CTAGGTAGGTATAAGAAGCCAACAACCACAGATATACCAACAATGGCTACA<br>GGAACTGCTCCACGCGCACTCATG
pRTL2/myc-Cb5Δp36TMD1	Fp1960	CTAGCACTGCCGTGGGGTTGGCCATCATACCTGGTATCCCGGCGATTGTGG<br>CAAACACACGTAAGAAGTAGT
	Rp1961	CTAGACTACTTCTTACGTGTGTTTGCCACAATCGCCGGGATACCAGGTATG<br>ATGGCCAACCCCACGGCAGTG
pRTL2/myc-Cb5Δp36TMD2	Fp1962	CTAGCATGCTCATTGGCCTGGCGGCGGCTGCAGCACTTGTTCCTATTTGTG<br>CTGGCATTGCGTACGCTACCCTACGTAAGAAGTAGT
	Rp1963	CTAGACTACTTCTTACGTAGGGTAGCGTACGCAATGCCAGCACAAATAGGA<br>ACAAGTGCTGCAGCCGCCGCCAGGCCAATGAGCATG
pRTL2/p36-mycΔ131-157	Fp1966	GGTACATGTGAGGTGGCAGGGCGTGTAGACATGAGTGCGCG
	Rp1967	CGCGCACTCATGTCTACACGCCCTGCCACCTCACATGTACC
pRTL2/p36-myc $K_{93}K_{94}R_{98}R_{101}\Delta G$	Fp412	GCTAAATATGATTGGTTGGCTGGAGGGGGTGTGGCTGGTGATTACGGCAC<br>TGCCGTGGGGTTGGCC
	Rp410	GGCCAACCCCACGGCAGTGCCGTAATCACCAGCCACACCCCCTCCAGCCA<br>ACCAATCATATTTAGC
pRTL2/p36-myc $R_{144}K_{151}\Delta G$	Fp2048	CTGAACATGCTTTCTGGATTCACACATGCGAGTGTAGGAGTACCGTACTTTTATCC
	Rp2049	GGATAAAAGTACGGTACTCCTACACTCGCATGTGTGAATCCAGAAAGCATGTTCAG
pRTL2/p36-myc $K_{134}K_{137}R_{144}K_{151}\Delta G$	Fp2098	GAGGTGGCAGGGGTTGCTGTAGGGCTGCCCGGGGCGCTGAACATGCTTTCTGGA
	Rp2099	TCCAGAAAGCATGTTCAGCGCCCCGGGCAGCCCTACAGCAACCCCTGCCACCTC
pSAT4/N Venus C1	Fp1905	CCGGCCATGGGCGGCGTGCAGCTCGCCGACCAC
	Rp1922	GAGCTGCACGCCGCCAGATCTGTCCTCGATGTTGTG
	Fp1941	GATCTGAACAAAAGTTGATTTCTGAAGAAGATCTGTCTCGAGCT
	Rp1946	ACTTGTTTTCAACTAAAGACTTCTTCTAGACAGAGC
pSAT4/N Venus N1	Fp1909	CCGGCCGGATCCTGATGGTGAGCAAGGGCGAGGAGCTG
	Rp1924	GGCGAGCTGCACGCCGCCTCTAGATTAGTCCTCGATGTTGTGGCG
	Fp1943	GGGCCCGGGGAACAAAAGTTGATTTCTGAAGAAGATCTGGG
	Rp1944	CGCCCGGGCCCCTTGTTTTCAACTAAAGACTTCTTCTAGACCCCTAG
pSAT4/C Venus C1	Fp1907	CCGGGCATGGGCGGCGTGCAGCTCGCCGACCAC
	Rp1908	CCGGCCAGATCTCTTGTACAGCTCGTCCATGCCGAG
	Fp1942	GATCTTACCCATACGACGTGCCAGACTACGCCTCTCGAGCT
	Rp1947	AATGGGTATGCTGCACGGTCTGATGCGGAGAGC
pSAT4/C Venus N1	Fp1911	CCGGCCGGATCCTGGGCGGCGTGCAGCTCGCCGACCAC
	Rp1912	CCGGCCTCTAGATTACTTGTACAGCTCGTCCATGCCGAG
	Fp1945	CGCCCGGGCCCATGGGTATGCTGCACGGTCTGATGCGGCCCTAG
	Rp1948	GGGCCCGGGTACCCATACGACGTGCCAGACTACGCCGG
pSAT4/N Venus-Tom20	Fp2088	CCGGCGGAATTCTATGGATACGGAAACTGAGTTCGATAG
	Rp2089	CCGGCGCCCGGGATTAACGAGGAGGAGAGACAGGCAC
pSAT4/N Venus-Tom20mut	Fp2274	CGAGCTCAAGCTTCGAATTCTGGCTTAGGCTCACAACCAATG
	Rp2275	CATTGGTTGTGAGCCTAAGCCAGAATTCGAAGCTTGAGCTCG
pSAT4/N Venus-Tom22	Fp2090	CCGGCGGAATTCTATGGCGCCTAAGAAAATCGGAGCC
	Rp2091	CCGGCGCCCGGGATTAGAGCATCGCACCGACCGGTGG

pSAT4/N Venus-Tom40	Fp2086	CCGGCGGAATTCTATGGCGGATCTTTTACCACCTCTTAC
	Rp2087	CCGGCGCCCGGGATTAACCAACTGTTAATCCGAAACC
pSAT4/Tom40-N Venus	Fp2086	CCGGCGGAATTCTATGGCGGATCTTTTACCACCTCTTAC
	Rp2087	CCGGCGCCCGGGATTAACCAACTGTTAATCCGAAACC
pCR2.1 TOPO/mtOM64	Fp2096	CCGGCGCTCGAGCATGTCGAATACGCTTTCTTTGATTC
	Rp2097	CCGGCGCCCGGGTATGTGTTTTCGGAGTCTCTTCTC
pSAT4/mtOM64mut-N Venus	Fp2272	CTCGATACAACTCTCGATCCCGGGGAACAAAAGTTGATT
	Rp2273	AATCAACTTTTGTTCCCCGGGATCGAGAGTTGTATCGAG
pSAT4/Tim14-N Venus	Fp2129	CCGGCGGAATTCATGGCGACACCATTTATAGCGGGGG
	Rp2284	CCGGGCCCCGGGAAAAGCGGATCCGCTGTTTTTAGTTTTGCC
pSAT4/p36-N Venus	Fp2094	CCGGCGGAATTCATGGAGGGTTTGAAGGCTGAGTCTAC
	Rp2095	CCGGGCCCCGGGTTTGACACCGAGGGATTCCTGGGAAC
pSAT4/p36-C Venus	Fp2094	CCGGCGGAATTCATGGAGGGTTTGAAGGCTGAGTCTAC
	Rp2095	CCGGGCCCCGGGTTTGACACCGAGGGATTCCTGGGAAC
pSAT4/p33-C Venus	Fp2176	GCTTCGAATTCATGGAGACCATCAAGAGAATGATTTGGCCTAAG
	Rp2095	CCGGGCCCCGGGTTTGACACCGAGGGATTCCTGGGAAC
pSAT4/βATPase-C Venus	Fp2092	GAATTCATGGCTTCTCGGAGGCAACAAGCCTTCAACAACGTCAATCGGC TCAACGTGG
	Rp2093	CCACGTTGAGCCGATTGACGTTGTTGAGAGGCTTGTTGCCTCCGAGAA GCCATGAATTC
pSAT4/porin-C Venus	Fp2130	CCGGCGGAATTCATGGTGAAAGGTCCCGGTCTCTAC
	Rp2131	CCGGGCCCCGGGAGGCTTGAGTGCGAGAGCCAATCC
pSAT4/ C Venus-Cb5	Fp1325	CGGGGCCATATGATGCCTACTCTCACCAAACTATTCACAATG
	Rp1324	GGCGCCGGATCCTCTAGACTACTTCTTACGTAGGTATAAGAA
pUC18/βATPase-GFP	FpRD1	GGGGGGGCTAGCATGGCTTCTCGGAGGCTTCTCGCC
	RpRD2	GGGGGGGCTAGCCCCGATCGAACCAGCGCCGG
